# Hepatocyte-Specific Deficiency of Endoplasmic Reticulum-Associated Degradation Induces Coordinated Innate–Adaptive Immune Responses

**DOI:** 10.3390/cells15171562

**Published:** 2026-08-28

**Authors:** Qingqing Liu, Junjie Wang, Haiying Zong, Hongqiong Ma, Mengyu Li, Menglei Wang, Dengfang Wu, Shujie Zheng, Han Wang, Qiaoming Long

**Affiliations:** 1 Jiangsu Key Laboratory of Neuropsychiatric Diseases and Cam-Su Mouse Genomic Resources Center, Medical College, Soochow University, Suzhou 215123, China; qqliu@suda.edu.cn (Q.L.);; 2 Center for Circadian Clocks, Soochow University, Suzhou 215123, China; wanghan@suda.edu.cn; 3 Jiangsu Key Laboratory of Drug Discovery and Translational Research for Brain Diseases, School of Basic Medical Sciences, Suzhou Medical College, Soochow University, Suzhou 215123, China

**Keywords:** liver injury, immune cell, ERAD, MAFLD, MASH, macrophage, immune homeostasis, T lymphocyte

## Abstract

**Highlights:**

**What are the main findings?**

- Hepatocyte ERAD deficiency expands multiple immune populations (CD8+ T cells, macrophages, monocytes, DCs) with functional reprogramming, and gives rise to two distinct TREM2-expressing macrophage subsets.
- Depletion of CD8+ T cells increased monocyte infiltration and aggravated liver injury, suggesting that CD8+ T cells exert a previously unrecognized protective role by restraining monocyte recruitment.

**What are the implications of the main findings?**

- These findings demonstrate coordinated innate–adaptive immune crosstalk and reveal that CD8+ T cell responses are not uniformly pathogenic in MASH.
- The identification of Trem2-high macrophages with lipid-handling and antigen-presenting functions, together with hepatoprotective CD8+ T cells, suggests that selectively preserving beneficial CD8+ T-cell states may be a promising therapeutic strategy for MASH.

**Abstract:**

Hepatic inflammation is a defining feature of Metabolic Dysfunction-Associated Steatohepatitis (MASH), yet the specific contributions of individual immune cell populations and their reciprocal interactions remain incompletely understood. In this study, we combined flow cytometry with single-cell transcriptomic profiling to characterize the hepatic immune landscape in a novel model of spontaneous MASH caused by hepatocyte-specific deficiency of endoplasmic reticulum-associated degradation (ERAD). Hepatic ERAD deficiency led to the expansion of multiple immune cell populations in the liver, including CD8+ T cells, macrophages, monocytes, and dendritic cells, accompanied by extensive functional reprogramming of both innate and adaptive immune compartments. Myeloid cells exhibited enhanced phagocytic activity and increased antigen processing and presentation, whereas CD8+ T cells displayed elevated proliferation capacity, DNA repair activity and cytotoxicity. Notably, two functionally distinct triggering receptor expressed on myeloid cells 2 (TREM2)-expressing macrophage subsets emerged during the progression of ERAD deficiency-induced MASH. Depletion of CD8+ T cells increased monocyte infiltration and aggravated liver injury, suggesting that CD8+ T cells exert a previously unrecognized protective role by restraining monocyte recruitment. Collectively, these findings reveal highly coordinated interactions between innate and adaptive cells during MASH progression and identify CD8+ T cells as potential regulators of monocyte infiltration and hepatic injury.

## 1. Introduction

Metabolic Dysfunction-Associated Steatohepatitis (MASH) is the progressive inflammatory form of Metabolic Dysfunction-Associated Fatty Liver Disease (MAFLD). It is characterized by hepatic steatosis accompanied by inflammation and hepatocellular injury and may progress to fibrosis, cirrhosis and hepatocellular carcinoma. MASH is closely associated with metabolic syndrome, and its major risk factors include obesity, type 2 diabetes, insulin resistance, hypertension, and dyslipidemia. Although its pathogenesis is multifactorial, insulin resistance is considered a central driver of excessive lipid accumulation and lipotoxicity in hepatocytes [1]. The resulting cellular stress and hepatocyte death activate inflammatory and fibrogenic pathways that promote disease progression [2,3].

Hepatic myeloid cells, particularly macrophages and dendritic cells (DCs), play central roles in the pathogenesis of MASH by acting as immune sentinels within the liver [4,5]. In response to metabolic stress and hepatocyte injury, including lipotoxic apoptosis and necrosis, these cells detect damage-associated molecular patterns (DAMPs) and phagocytose cellular debris [6]. Activated macrophages subsequently produce proinflammatory cytokines and chemokines, such as TNFα, IL-6 and CCL4, thereby recruiting additional immune cells and establishing a sustained inflammatory microenvironment [5,7]. DCs further connect innate and adaptive immunity through antigen processing and presentation to T cells [8,9]. Hepatic macrophage and DC populations expand substantially in both patients with MASH and experimental disease models [10,11]. Notably, depletion of macrophages or DCs can exacerbate hepatic inflammation and injury, indicating that these cells may exert context-dependent functions that extend beyond inflammation to include the clearance of damaged cells, tissue repair, and restoration of hepatic homeostasis [11,12].

Accumulating evidence from patients and animal models indicates that MASH progression is accompanied by marked increases in peripheral and intrahepatic B, CD4+, and CD8+ T cells [13,14]. Moreover, single-cell transcriptomic and secretome analyses have revealed an extensively interconnected signaling network among hepatocytes, vascular cells, and innate and adaptive immune populations that contribute to MASH development [15]. Nevertheless, the specific functions of individual adaptive cell populations and their interactions with hepatic myeloid cells during MASH initiation and progression remain scarcely understood [13,14].

The endoplasmic reticulum-associated degradation (ERAD) machinery removes misfolded proteins from the ER and constitutes a key mechanism of unfolded protein response (UPR) [16]. SEL1L is an essential component of the HRD1-containing E3 ubiquitin ligase complex and facilitates ERAD function [16]. To investigate how ERAD deficiency in hepatocytes shapes hepatic immunity, we generated hepatocyte-specific Sel1l knockout (HKO) mice by crossing floxed Sel1l mice (Sel1l^F/F^) with Alb-Cre transgenic mice [17].

In this study, we combined flow cytometry with single-cell RNA sequencing (scRNA-seq) to characterize the hepatic immune landscape in a spontaneous MASH mouse model caused by hepatocyte-specific deficiency of endoplasmic reticulum-associated degradation (ERAD). Our findings reveal coordinated remodeling of the innate and adaptive compartments during MASH progression and identify a potential role for CD8+ T cells in limiting monocyte infiltration and liver injury.

## 2. Materials and Methods

### 2.1. Mice

All mouse strains used in this study are listed in Table 1 Mice with floxed Sel1l alleles (Sel1l^F/F^) were previously described [16]. To generate hepatocyte-specific Sel1l knockout (HKO) and wild-type (WT) control mice, Sel1l^F/F^ mice were first crossed to transgenic mice expressing Cre recombinase under the control of the mouse albumin gene proximal promoter (Alb-Cre) [18]. The resulting Sel1l^F/+^; Alb-Cre mice were backcrossed with Sel1l^F/F^ mice to generate Sel1l^F/F^; Alb-Cre mice. Next, Sel1l^F/F^; Alb-Cre mice were crossed with Sel1l^F/F^ mice to generate HKO (Sel1l^F/F^; Alb-Cre) and WT (Sel1l^F/F^) littermate control mice. Mice were housed under standard conditions with a 12 h/12 h light/dark cycle, temperature control at 22–24 °C, humidity control at 60%, unrestricted food and water availability, and daily inspection of the mice. All mice were housed in a specific pathogen-free (SPF) environment at the Laboratory Animal Center, and all animal procedures were approved by the Ethics Committee of Soochow University (approval number: SUDA20260520A09).

### 2.2. Immunological Depletion of CD8+ T Cells

Two-month-old HKO mice were randomly divided into control and antibody treatment groups and injected (200 μg/mouse/injection) intraperitoneally (IP) with IgG2b (*n* = 9) or a monoclonal antibody (mAb) against CD8α (*n* = 10) (Bio X Cell), respectively, every 3 days for 1 month. The mAb and IgG-treated mice were monitored for growth and behavior abnormalities. Two days after the last injection, mice were sacrificed and processed for biochemical and histological assessments of liver injury and hepatic immune cell composition. Bulk RNA sequencing was performed on RNA extracted from the livers of mice in the IgG (*n* = 3) and mAb (*n* = 3) groups. The raw sequence data reported in this paper have been deposited in the Genome Sequence Archive [19] in National Genomics Data Center [20], China National Center for Bioinformation/Beijing Institute of Genomics, Chinese Academy of Sciences, and are publicly accessible at https://ngdc.cncb.ac.cn/gsa/browse/CRA039846 (accessed on 23 August 2026).

### 2.3. Isolation and Flow Cytometry Analysis of Hepatic Immune Cells

Mice were anesthetized by CO_2_ inhalation, and their livers were immediately dissected and cut into 2 mm pieces. Mouse livers were minced and digested in DMEM containing 0.02% Collagenase IV and 4 U/mL DNase I at 37 °C for 30 min. The digest was filtered through a 70 μm cell strainer, centrifuged at 50× *g* for 2 min and then at 400× *g* for 5 min. The cell pellet was washed with PBS, incubated in red blood cell lysis buffer for 5 min at room temperature, and collected as the NPC fraction. The RBC-free cell pellets (i.e., the NPCs) were washed once with PBS and resuspended in a flow cytometry staining buffer (65-0865-14, eBioscience, Carlsbad, CA, USA) at roughly 1 × 10^7^ cells/mL. For flow cytometry analysis, 100 μL of immune cell-containing staining buffer was mixed with 1 μg of anti-mouse CD16/CD32 antibody and incubated at 4 °C for 10 min for Fc blocking. After centrifugation (300× *g* for 5 min at 4 °C) to remove the antibodies, cells were resuspended in 100 μL staining buffer containing the fixable viability dye eFluor780 (1 μL/1 mL) and incubated in darkness for 30 min at 4 °C. Cells were then washed twice with 1 mL cold staining buffer and resuspended in the same buffer containing fluorescence-tagged antibodies (Table 2). Flow cytometry was performed on a Beckman Cytoflex flow cytometer (Beckman Coulter Life Sciences, Indianapolis, IN, USA), and data were analyzed using FlowJo10.8.1. Flow cytometry data were analyzed using a hierarchical gating strategy. Debris and doublets were excluded based on FSC-A/SSC-A and FSC-H/FSC-A plots, respectively, and dead cells were removed by staining with the Fixable Viability Dye eFluor™ 780. CD45+ cells were identified as leukocytes and analyzed using three parallel gating branches. F4/80+ cells were classified as total macrophages and subdivided into Kupffer cells (KCs, CD11b^−^) and myeloid-derived macrophages (MDMs, CD11b+). CD11c+ cells were classified as dendritic cells (DCs) and activated DCs (MHCII+). CD19+ cells within the CD11b- fraction were identified as B cells, whereas CD3+ cells within the CD19- fraction were identified as T cells, which were further subdivided into CD4+ and CD8+ T cells. Within the CD11b+ fraction, Ly6C+ and Ly6G+ cells were identified as monocytes and granulocytes, respectively. Representative gating strategies for the identification of hepatic immune cell populations are shown in Figure S7.

### 2.4. Serum Biochemical Analysis

Mice were fasted overnight and anesthetized the next day with isoflurane. Blood was collected from the orbital sinus into heparin sodium-treated 1.5 mL centrifuge tubes and placed on ice for 1 h. After centrifugation to remove blood cells, the plasma was analyzed using a Hitachi 7100 automatic biochemical analyzer (Hitachi High‑Technologies Corporation, Tokyo, Japan). The serum parameters monitored include Alanine Aminotransferase (ALT), Aspartate Aminotransferase (AST), Lactate Dehydrogenase (LDH), Alkaline Phosphatase (ALP), Total Bilirubin (TBIL), Uric Acid (UA), High-density Lipoprotein (HDL) and Low-Density Lipoprotein Cholesterol (LDL).

### 2.5. RNA Isolation and Quantitative RT-PCR

Total RNA was extracted from mouse liver tissue or isolated primary hepatocytes using RNAiso Plus (Takara, Kusatsu, Japan) according to the manufacturer’s instructions. After DNase I treatment, 1 µg of RNA was used in a reverse transcription reaction to generate 1st strand cDNA using the HiScript III 1st Strand cDNA Synthesis Kit (Vazyme, Nanjing, China). Quantitative PCR was performed using SYBR Green (Vazyme) and predesigned gene-specific primers (Table 3) on a ViiA7 Real-Time PCR system (Life Technologies, Carlsbad, CA, USA). Fold changes in mRNA levels were determined using the comparative threshold cycle (Ct) method, and target gene transcription was normalized to that of β-Actin. The primers used are listed in Table 3.

### 2.6. Plasmids, Cell Transfection and Fluorescent Imaging

Plasmids pCMV-mCherry-TGN38 and pCMV-mCherry-RAB5A were purchased from Miao-Ling Plasmid (Wuhan, China). Plasmid pPBH-TREtight-Trem2-EGFP was generated by cloning the open reading frame (ORF) region of mouse Trem2 cDNA (NCBI accession: NM_018965.4) into NheI-linearized pPBH-Teton-Nango vector (kindly provided by Dr. Wensheng Zhang) via homologous recombination.

Cell lines used in this study are listed in Table 1. HepG2 cells were cultured in DMEM medium supplemented with 15% FBS, 100 U/mL penicillin,100 μg/mL streptomycin at 37 °C in a humidified CO_2_ incubator and transfected using Lipofectamine 3000 (Thermo Fisher Scientific, Carlsbad, CA, USA). For stable cell line generation, HepG2 cells were co-transfected with pPBH-TREtight-Trem2-EGFP and a transposase plasmid at a 3:1 ratio. 48 h post-transfection, cells were selected with Hygromycin until no further cell death was observed. Trem2-EGFP fusion protein expression was induced by treating the cells with 1 μg/mL doxycycline hydrochloride (Dox). Green or red fluorescent images were acquired on a FluoviewFV1000 Olympus (Tokyo, Japan) confocal microscope and analyzed by Image J (1.53t) software.

### 2.7. Single-Cell RNA Sequencing

Hepatic NPC suspensions from WT (*n* = 1) and HKO (*n* = 1) mice were prepared as described in Section 2.3. Briefly, livers were minced and digested with Collagenase IV and DNase I, filtered through a 70 μm cell strainer, and subjected to differential centrifugation (50× *g* for 2 min and then 400× *g* for 5 min) to remove hepatocytes and other non-immune cell aggregates, followed by lysis of red blood cells. The residual hepatocytes in the obtained NPC fraction were identified using hepatocyte-specific markers (e.g., Alb and Ttr) during initial scRNA-seq processing and excluded from all downstream immune-cell analyses. For each genotype, cells from four genotype-matched mice were pooled and loaded onto a 10× Chromium controller to generate single-cell Gel Bead-In-Emulsions (GEMs). The GEMs were then subjected to library construction using the Chromium^TM^ Single Cell 3′Reagent Kit (version 3.1) (10× Genomics, Pleasanton, CA, USA) according to the manufacturer’s instructions. Library construction and RNA sequencing were completed by Gene Denovo Biotechnology Co., Ltd. (Guangzhou, China) as described [21,22]. The raw sequence data reported in this paper have been deposited in the Genome Sequence Archive [19] in the National Genomics Data Center [20], China National Center for Bioinformation/Beijing Institute of Genomics, Chinese Academy of Sciences, and are publicly accessible at https://ngdc.cncb.ac.cn/gsa/browse/CRA039675 (accessed on 23 August 2026).

### 2.8. scRNA-Seq Data Quality Control and Normalization

Barcode processing, data quality control and normalization were performed using the Cell Ranger Single Cell Software v3.1.0 (10× Genomics, Pleasanton, CA, USA). Briefly, raw data from the sequencer were demultiplexed using cellranger mkfastq (10× Genomics, Pleasanton, CA, USA), and reads were aligned to the mouse reference genome (mm10) using the STAR aligner implemented in Cell Ranger (v3.1). Quality control was performed as follows: cells with fewer than 500 unique molecular identifiers (UMIs), fewer than 500 detected genes, more than 4000 detected genes, or more than 10% of UMIs mapping to mitochondrial genes were excluded. Potential doublets were identified using DoubletFinder (v2.0.3) and removed from downstream analysis. After QC and doublet removal, a total of 20,055 cells (8390 from WT and 11,665 from HKO cells) were retained. The raw UMI counts were normalized by dividing each cell’s counts by its total UMI count, multiplying by a scale factor of 10,000, adding a pseudo-count of 1, and applying a log transformation.

### 2.9. Cell Clustering and Visualization

Data clustering was performed using the Seurat R package v4.0.4. Briefly, filtered and normalized WT and HKO datasets were integrated after canonical correlation analysis-based reduction in batch effects. The integrated data were further normalized by Z-score and then subjected to principal component analysis (PCA) to reduce dimensionality. Subsequently, the enriched PCs with low *p*-value genes were used in a shared-nearest neighbor (SNN) graph approach to cluster cells. The Find-Cluster tool employing the Louvain algorithm was used to group cells into different subsets according to their expression levels. Single-cell subgroup classification results were visualized by t-distributed Stochastic Neighbor Embedding (t-SNE) using the Loupe Cell Browser software. For each cell cluster, genes showing differential expression and with known functions were identified.

### 2.10. Cell–Cell Communication and Receptor-Ligand Analysis

CellPhoneDB v5.0.0 identifies significant ligand–receptor pairs by requiring ligand expression in sender cells and receptor expression in receiver cells (each >10% of cells). Significance is assessed via permutation tests (1000 permutations, *p* ≤ 0.05), and pairs are ranked by the product of mean expression levels.

### 2.11. Single-Cell Pseudo-Time Analysis

T cell subsets were identified based on gene expression levels. Subsequently, pseudotime analysis was separately performed on the CD8T and CD4T clusters to analyze the temporal order corresponding to each cluster. Single-cell trajectory analysis was performed with the Monocle v2.10.1 package [23]. Briefly, key differentially expressed genes (DEGs) related to the development and differentiation processes were identified by performing differential gene tests and subsequently used as markers to define cellular progress. Data dimension reduction was performed using DDRTree v0.1.5, and cells were ordered in pseudotime with the order-cells function. The trajectory was visualized in a two-dimensional tree-like structure by running the plot cell trajectory function.

### 2.12. Gene Expression Program (GEP) Analysis

Gene expression programs underlying cellular activities in hepatocytes, myeloid and T cells were identified using the consensus non-negative matrix factorization (cNMF) method [24]. Briefly, normalized cell type-specific expression data from WT and HKO mice were integrated and used as input to run non-negative matrix factorization (NMF) analysis to identify clusters of highly similar components inferred as GEPs. This procedure was repeated multiple rounds for each cell type, and a consensus k-value (number of GEPs) was selected that provides a reasonable trade-off between error and stability. Non-negative least squares (NNLS) were used to calculate the activity of NMF transcription programs in each cell based on the first 100 weighted genes of the GEP [25]. Subsequently, Student’s *t*-test or Mann–Whitney test statistical analyses were performed to compare GEP activity values between WT and HKO cells, followed by multiple testing correction, and *p* < 0.05 was defined as statistically different. The top 30 genes of each GEP that showed significant activity differences between WT and HKO cells were used in GO, KEGG or Reactome analysis to identify the biological functions associated with the GEP [26,27]. Finally, tSNE plots generated with the ggplot2 package were used to visualize the spatial distribution of the statistically different GEPs in cell subtypes.

### 2.13. Statistical Analysis

Differences between compared groups were evaluated by performing Student’s *t*-test or two-way repeated ANOVA using Graphpad Prism (8.4.2). Data are presented as mean ± standard error, and *p* < 0.05 was considered significant.

### 2.14. Analysis of Publicly Available Human MASH Single-Nucleus RNA-Sequencing Data

To assess translational relevance, we re-analyzed a public human liver snRNA-seq dataset (GEO: GSE202379; Gribben et al., 2024) [28]. The cohort spans the MAFLD/MASH spectrum. It includes 59 samples from 47 patients (healthy, *n* = 6; NAFLD, *n* = 7; NASH, *n* = 27; NASH with cirrhosis, *n* = 4; end-stage disease, *n* = 15). Normalized count matrices were downloaded from GEO and processed in Python v3.12 (pandas 3.0.5, NumPy2.5.1, SciPy 1.18.0). Hepatocytes were defined as ALB-expressing cells. A composite UPR score was calculated as the mean log-normalized expression of eight core UPR/ERAD genes (HSPA5 (BiP), ERN1 (IRE1α), XBP1, DDIT3 (CHOP), ATF4, EIF2AK3 (PERK), ATF6, SEL1L) across ALB+ cells. Immune cell fractions were quantified by lineage markers: CD45+ cells (PTPRC), T cells (CD3D/CD3E), CD8+ T cells (CD8A/CD8B), CD4+ T cells (CD3D/CD3E plus CD4), B cells (CD79A/MS4A1), NK cells (NKG7/KLRD1), Kupffer cells (C1QC and MARCO), macrophages (C1QC/CD68/CD163/MARCO), monocytes (LYZ plus CD14/FCGR3A/VCAN), dendritic cells (CD1C/CLEC9A/LILRA4) and neutrophils (S100A8/S100A9). Associations were tested with two-sided Spearman correlations, at the sample level (*n* = 59) and at the patient level (*n* = 47) after averaging samples from the same patient. Diagnostic groups were compared with Kruskal–Wallis tests. Disease-stage trends were assessed with Spearman correlations against ordinal stage. *p* values were adjusted across immune cell populations using the Benjamini–Hochberg procedure.

## 3. Results

### 3.1. Hepatocyte ERAD Deficiency Triggers Extensive Migration of Myeloid and Lymphoid Cells into the Liver

**Figure 1:**
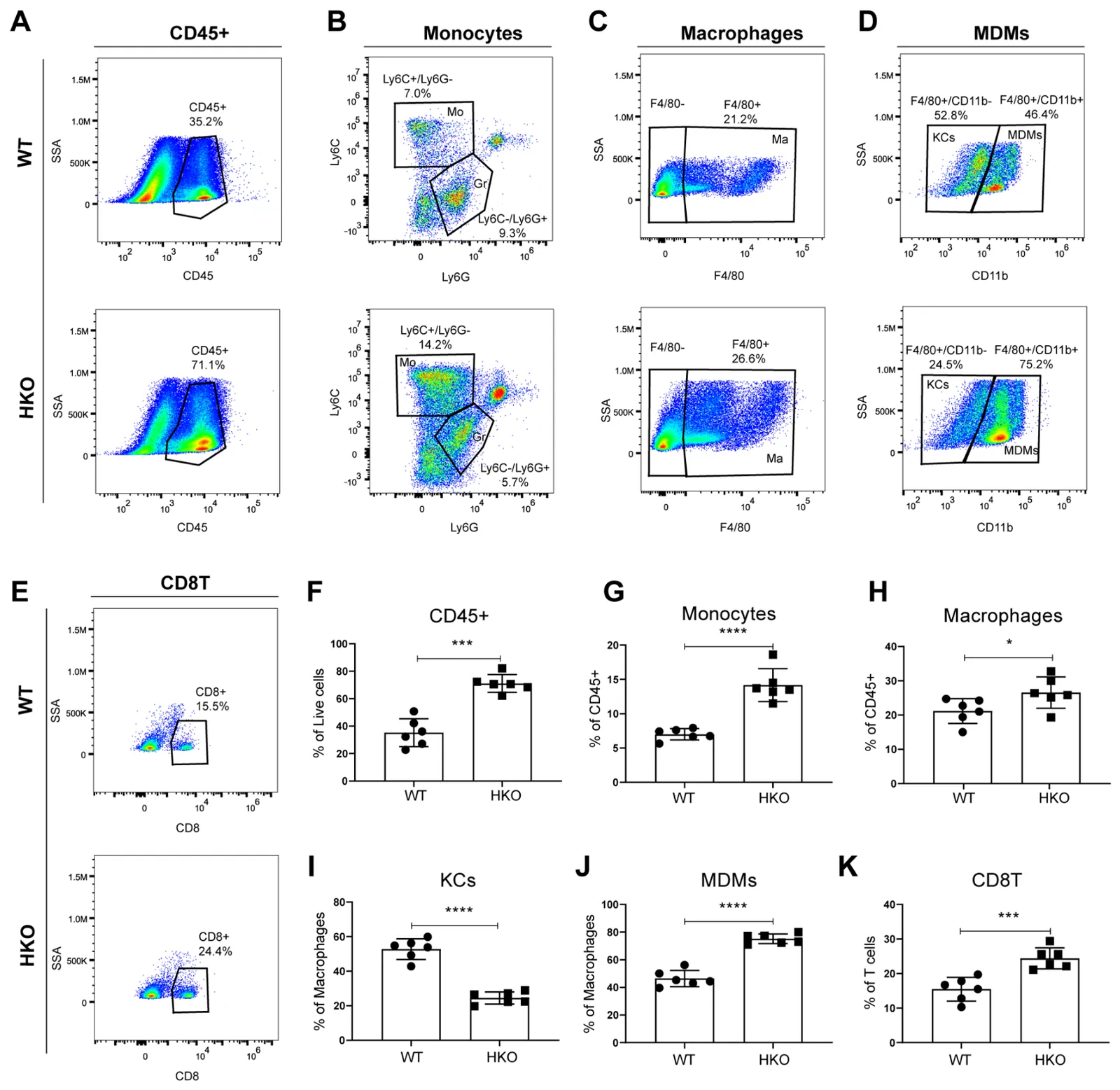
Hepatocyte-specific ERAD deficiency triggers significant expansion of both innate and adaptive immune cells in the liver. (**A**–**E**) Flow cytometry gating for quantification of hepatic total immune cells (**A**), monocytes (**B**), macrophages (**C**), KCs and MDMs (**D**) and CD8+ T cells (**E**) in WT vs. HKO mice. (**F**–**K**) Quantification of relative abundance of total immune cells (**F**), monocytes (**G**), total macrophages (**H**), KCs (**I**), MDMs (**J**) and CD8+ T cells (**K**) in WT vs. HKO mice. All data are presented as means ± SEM (*n* = 6). ns, not significant; * *p* < 0.05, *** *p* < 0.001, **** *p* < 0.0001 by two-tailed Student’s *t* test.

We recently characterized the metabolic phenotype of mice with hepatocyte-specific knockout of Sel1l, a key component of the endoplasmic reticulum-associated degradation (ERAD) complex. These mice, referred to as HKO, develop spontaneous MASH due to perturbed mitochondrial function in hepatocytes (Figure S1). At 3 months, HKO mice showed liver injury (Figure S1A–D) with normal liver weight and histology. ER stress markers were significantly upregulated in HKO livers, as shown by elevated IRE1α and p-PERK levels and increased ATF6 and CHOP mRNA expression (Figure S1E–G). In addition, hepatocytes exhibited altered mitochondrial morphology and disrupted endoplasmic reticulum structure (Figure S1H). By 6 months, they developed increased nuclear enlargement (Figure S1I), granulocyte infiltration (Figure S1I), and lipid deposition (Figure S1J). To assess the immune responses to ERAD deficiency-mediated hepatocyte damage, we first used flow cytometry to analyze the total immune content (CD45+ cells) and relative abundances of major immune cell types (Monocytes, Macrophages, DCs, NK, CD4 and CD8 T cells) in the liver of HKO mice. CD45+ cells accounted for 35.2% of the non-parenchymal component in WT liver but increased to 71.1% in HKO liver (Figure 1A,F), indicating a significant expansion of the hepatic immune compartment in the knockout mice. The percentages of B, T, CD4+ T and DC among the CD45+ cell population were not different between WT and HKO (Figure S2A–K), however, those of monocytes (Figure 1B,G), macrophages (Figure 1C,H) and CD8+ T cells (Figure 1E,K) were significantly increased in HKO liver. When hepatic macrophages were separated into resident Kupffer cells (KCs) and myeloid-derived macrophages (MDMs) fractions, a sharp fall in KC abundance (52.8 to 24.5%) and a marked increase in MDM abundance (46.4 to 75.2%) were observed in HKO mice (Figure 1I,J). These flow cytometry results indicate that hepatocyte ERAD deficiency triggers an extensive remodeling of the hepatic immune compartment, with a sharp decrease in liver-resident macrophages and expansion of myeloid and CD8+ T cells.

### 3.2. Hepatocyte ERAD Deficiency Alters the Overall Intrahepatic Immune Landscape and Intercellular Signaling Network

**Figure 2:**
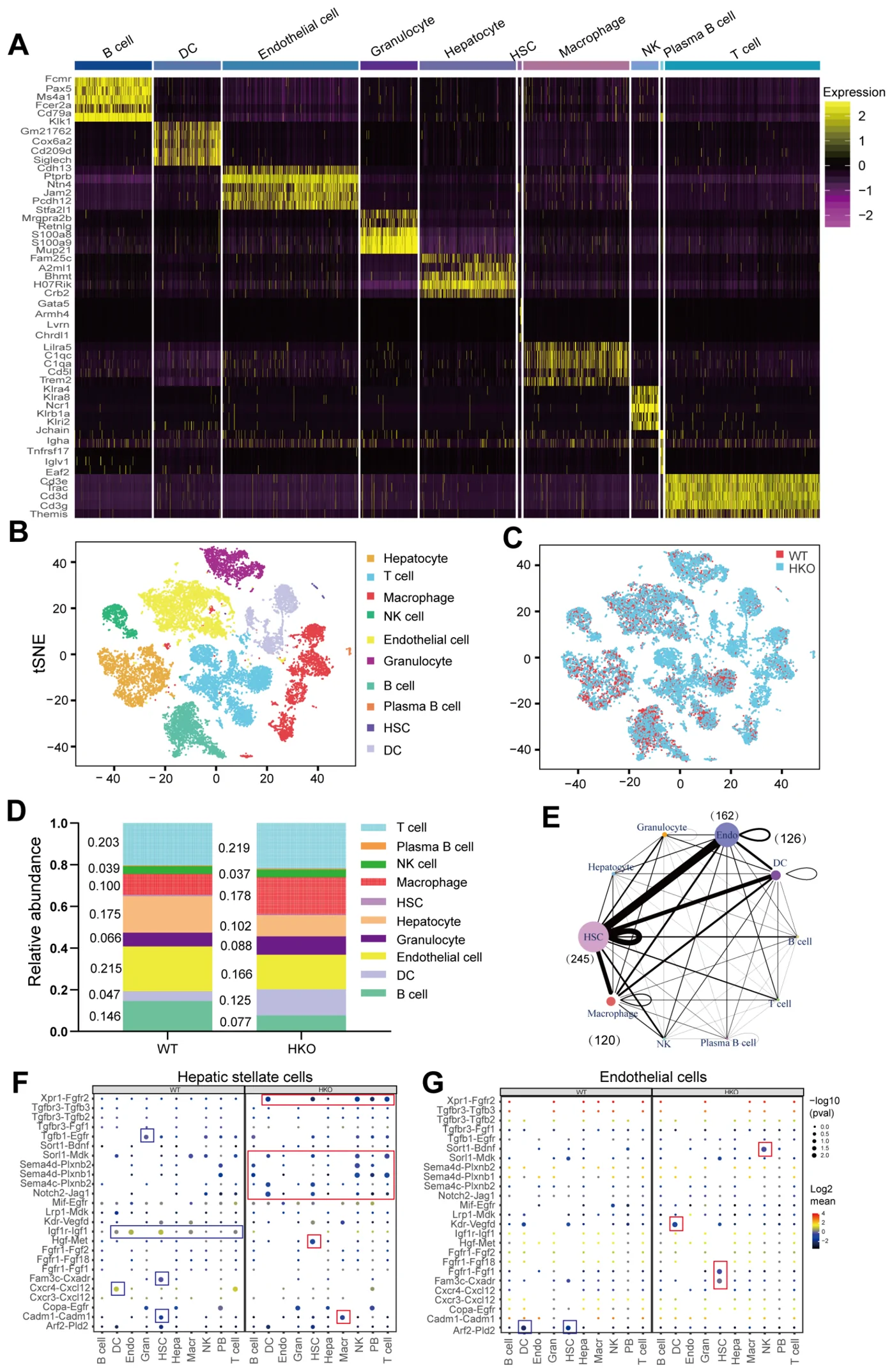
Hepatocyte ERAD deficiency alters the overall hepatic immune landscape and intercellular crosstalk. (**A**) Heatmap showing cell-specific expression of the top 5 genes in each of the defined hepatic cell types. (**B**,**C**) tSNE plots showing the hepatic cell populations defined using integrated (**B**) and separated (**C**) WT and HKO scRNA-seq data. (**D**) Stacked bar charts showing relative abundances of the defined hepatic cells in WT and HKO liver. (**E**) Network visualization of signaling hubs in the liver. (**F**,**G**) Bubble charts showing altered ligand–receptor connectivity in HSC (**F**) and endothelial cells (**G**) of HKO vs. WT liver. The up- and down-regulated pathways are marked by red and blue rectangles, respectively.

To obtain a more detailed profile of the hepatic immune landscape in HKO mice, we isolated hepatic non-parenchymal cells (NPCs) from WT and HKO mice and subjected them to scRNA-seq. After quality control, a total of 8390 WT and 11,665 HKO cells were transcriptionally profiled. Unsupervised clustering analysis using integrated WT and HKO transcriptome data defined 10 significantly different cell types, each showing unique expression of marker genes (Figure 2A). Hepatocyte-derived cells, identified by hepatocyte marker genes such as Alb, Apoa2 and Ttr, were excluded from all downstream immune-cell analyses (Section 2.7). Lineage-specific biomarker-based functional annotation showed that the 10 cell types include hepatocytes, macrophages, granulocytes, endothelial cells, natural killer cells (NK), dendritic cells (DC), hepatic stellate cells (HSC), plasma B cells, and B and T lymphocytes (Figure 2A,B). Coloring of WT and HKO cells on the obtained tSNE plot allowed for a semi-quantitative comparison of the relative abundance of each hepatic immune cell type in WT and HKO mice (Figure 2C). Three myeloid cell types, including macrophages (10 to 17.8%), dendritic cells (4.7 to 12.5%), granulocytes (6.6 to 8.8%) and T lymphocytes (20.3 to 21.9%), were increased, while endothelial and B cells were decreased in abundance in HKO liver (Figure 2D). These findings are consistent with the conclusion based on flow cytometry analysis that hepatocyte ERAD deficiency triggered remodeling of the overall hepatic immune compartment, with substantial expansion of macrophage, DC and T cell populations.

Intercellular crosstalk via ligand and receptor signaling plays a fundamental role in maintaining liver functions and tissue homeostasis [29]. Thus, we next assessed the differential expression of transcripts encoding secreted factors (the secretome) in ERAD-deficient hepatocytes by searching a reference secretome database [30]. Of the 978 cataloged secreted-membrane proteins, 245 were detected in hepatic stellate cells (HSCs), while 162, 120 and 126 were detected in endothelial cells, macrophages and DCs, respectively (Figure 2E), supporting the claim that HSCs, endothelial cells and macrophages function as key hubs for hepatic paracrine and autocrine signaling [15]. To assess how hepatocyte ERAD deficiency impacts secretome gene expression, we examined the expression of the top 25 differentially expressed ligand-receptor genes. Six ligand-receptor genes, including Xpr1-Fgfr2, Sorl1-Mdk, Sema4d-Plxnb2, Sema4d-Plxnb1, Sema4c-Plxnb2 and Notch2-Jag1, were broadly upregulated in the HSC signaling hub (Figure 2F), while four gene pairs, including Sort1-Bdnf, Lrp1-Mdk, Fgfr1-Fgf2 and Fgfr1-Fgf18, were selectively increased in the endothelial signaling hub (Figure 2G). Together, these findings suggest that hepatocyte ERAD deficiency alters the hepatic immune landscape and cell–cell communications through ligand–receptor signaling in the liver.

### 3.3. MASH-Associated Myeloid Cells Show Enhanced Phagocytosis, Antigen Processing and Presentation Activities

Myeloid cells have long been recognized as sentinels of the liver and are usually the first to respond to invading hepatic pathogens [31]. To understand how myeloid cells react to ERAD deficiency-mediated hepatocytic damage, we analyzed the transcriptome profiles of these cells. Unsupervised clustering analysis using integrated WT and HKO myeloid cell scRNA-seq data yielded 11 myeloid cell clusters. Of these, six (C01–06) were classified as macrophages, four (C07–10) as dendritic cells and one (C11) as granulocytes (Figure S3A,B). All myeloid clusters, except for C04, C06 and C10, were substantially expanded in the liver of HKO mice (Figure S3C,D). These findings are in line with the flow cytometry results and suggest that the hepatic myeloid compartment undergoes significant expansion in response to ERAD deficiency-induced hepatocyte damage.

**Figure 3:**
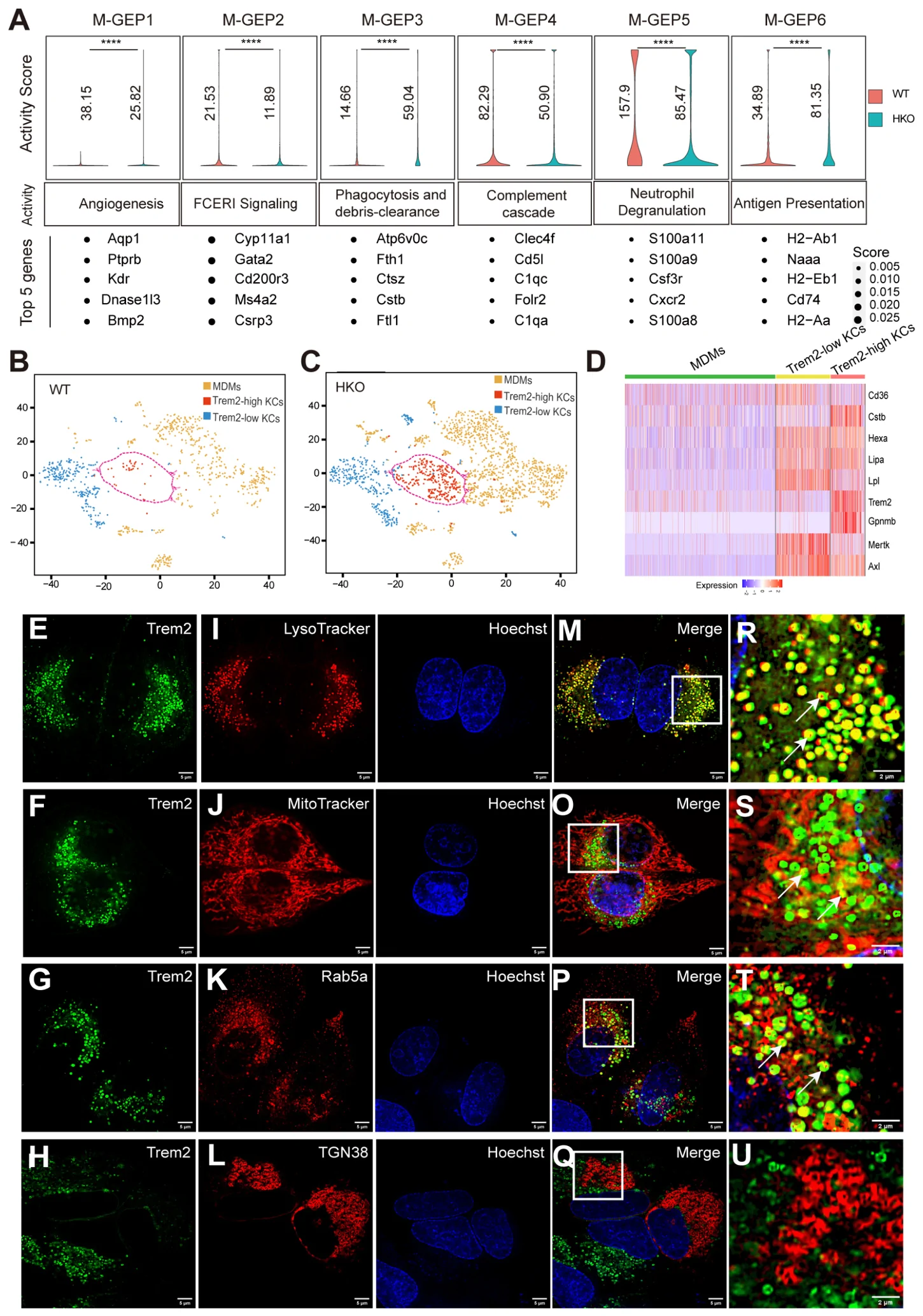
MASH-associated myeloid cells exhibit exacerbated activities in phagocytosis, antigen processing and presentation. (**A**) Violin plots showing altered myeloid cell gene expression programs (M-GEPs) in HKO Vs. WT livers (numbers represent GEP activity scores, **** *p* < 0.0001 by two-tailed Student’s *t* test). GEP names and the top 5 genes are indicated underneath. (**B**,**C**) tSNE plots showing spatial distribution of MDMs, Trem2-low and Trem2-high-expressing (dashed red lines) macrophages in WT (**B**) Vs. HKO (**C**) livers. (**D**) Heatmap showing the expression of lipid metabolism- and phagocytosis-related genes in MDMs, Trem2-low KCs and Trem2-high KCs macrophage subsets. (**E**–**U**) Representative immunofluorescence images showing spatial distribution of ectopically expressed Trem2-GFP (**E**–**H**) and LysoTracker (**I**,**M**,**R**), MitoTracker (**J**,**O**,**S**), Rab5a-mCherry (**K**,**P**,**T**) and TGN38-mCherry (**L**,**Q**,**U**). (**R**–**U**) are magnified views of the white square-marked areas in (**M**,**O**–**Q**), respectively. White arrows indicate colocalization. Nuclei are stained with DAPI (blue).

Next, we assessed how myeloid cells functionally respond to ERAD deficiency-mediated hepatocyte damage. For this, we used consensus non-negative matrix factorization (cNMF), a method for identifying gene expression programs underlying cellular activities [24], to generate myeloid-specific gene expression programs (M-GEPs). cNMF analysis yielded six distinct myeloid GEPs with differential activities between WT and HKO myeloid cells (Figure 3A, upper panels). Functional examination of the top 30 genes in each GEP revealed that M-GEP3 exhibits functions related to phagocytosis and debris clearance, which are associated with homeostatic or immunosuppressive phenotypes. M-GEP6 is related to antigen processing or presentation, while M-GEP 1, 2, 4 and 5 are associated with angiogenesis, FCER1 signaling, complement cascade and neutrophil degranulation, respectively (Figure 3A, middle and lower panels). Of note, M-GEP1, 2, 4 and 5 showed decreased activity, whereas M-GEP3 and 6 showed increased activity in HKO liver (Figure 3A, top panels). These results suggest that, in response to hepatocyte ERAD deficiency, the antigen processing and presenting activities of myeloid cells are enhanced, whereas other myeloid cell activities such as neutrophil degranulation, complement cascade and angiogenesis promotion are weakened.

Triggering receptor expressed on myeloid cells 2 (Trem2) is upregulated in a subset of hepatic macrophages during MASH pathogenesis in mice and human patients [15], and its genetic deletion exacerbates hepatic steatosis and inflammation [32]. Thus, we next asked whether the Trem2+ macrophage population also existed in the HKO liver. Hepatic macrophages were first separated into Kupffer cells (KCs) and myeloid-derived macrophages (MDMs) sub-populations based on known KC (Cd5l, Cd9, Mmp12, Gpnmb and Clec4f) and MDM (Ccr2, Cd11b, Ly6c2, Ms4a4c and Gpr141b) markers [15]. Notably, Trem2 expression was detected in KCs but not in the MDMs; moreover, KCs appeared as two equally sized subsets: Trem2-high- and Trem2-low-expressing cells in HKO but not in WT livers (Figure 3B,C). Both Trem2-high-expressing and Trem2-low-expressing KCs expressed a mixed set of functional biomarkers, including lipid-associated (LA) and phagocytosis-related (Figure 3D), proinflammatory (M1) and anti-inflammatory (M2) macrophage markers (Figure S4A). Gene ontology analysis revealed that the differentially expressed genes (DEGs) in Trem2-high-expressing cells were mainly enriched in antigen processing and presentation or immune response pathways, while those in Trem2-low-expressing cells were mostly associated with GTPase or hydrolase activity regulation pathways (Figure S4B,C). These data indicate that both Trem2-low and Trem2-high KCs are engaged in lipid metabolism and phagocytosis (Figure S4E); however, Trem2-high KCs additionally upregulate antigen-processing and presentation pathways, suggesting a potential link between lipid handling and adaptive immune activation.

Although myeloid-specific Trem2 knockout mice have been characterized [32], how TREM2 is involved in suppressing MASH progression is still unknown. To gain insights into the mechanistic roles of Trem2, we examined the intracellular distribution of ectopically expressed GFP-TREM2 fusion protein in HepG2 cells, a system chosen for its transfection tolerance after attempts in macrophage lines proved unsuccessful (see Section 4) in relation to major functional organelles (Figure 3E–H). Confocal fluorescent imaging revealed that GFP-TREM2 co-localizes exclusively with Lysotracker (Figure 3E–R), while only partially with MitoTracker (Figure 3F–S) and the co-expressed endosomal marker mCherry-tagged RAB5A (Figure 3G–T). Of note, GFP-TREM2 showed no co-localization with the co-expressed Golgi apparatus marker TGN38 (Figure 3H–U). These findings indicate that TREM2 is likely engaged in engulfing and transporting phagocytosed cells or lipids to lysosomes for hydrolytic degradation, and macrophages upregulate Trem2 expression to promote their lysosomal functions during MASH progression. Overall, these analyses provide a comprehensive and quantitative assessment of transcriptional changes in hepatic myeloid cells in response to ERAD-deficiency-induced hepatocyte damage.

### 3.4. MASH-Associated T Cells Show Increased Proliferation, DNA Repair and Effector Function but Suppressed Memory Activity

Having characterized the myeloid compartment, we next examined T lymphocytes, which scRNA-seq identified as another expanded population in HKO livers. Immune surveillance by T cells has been shown to play an essential role in immune defense against viral and bacterial infections [33,34]. However, how T lymphocytes react to metabolically induced liver damage remains unclear. To address this question, we compared the transcriptome data of T cells in WT and HKO mice. Unsupervised clustering using integrated WT and HKO transcriptome data defined hepatic T cells into 6 clusters: two CD4+ (C01, C02), three CD8+ (C03, C04 and C05) and one double-negative T (DNT) (C06) (Figure S5A,B); the gene annotation results are listed in Table S1. tSNE analysis of WT and HKO transcriptome data revealed that clusters 01, 03 and 05 remained unchanged in cell numbers; clusters 02, 04 and 06 were markedly expanded in HKO liver (Figure S5C,D). These findings suggest that hepatocyte ERAD deficiency induces expansion of certain subsets of T lymphocytes and explain why flow cytometry analysis revealed no significant change in T cell numbers between WT and HKO livers.

**Figure 4:**
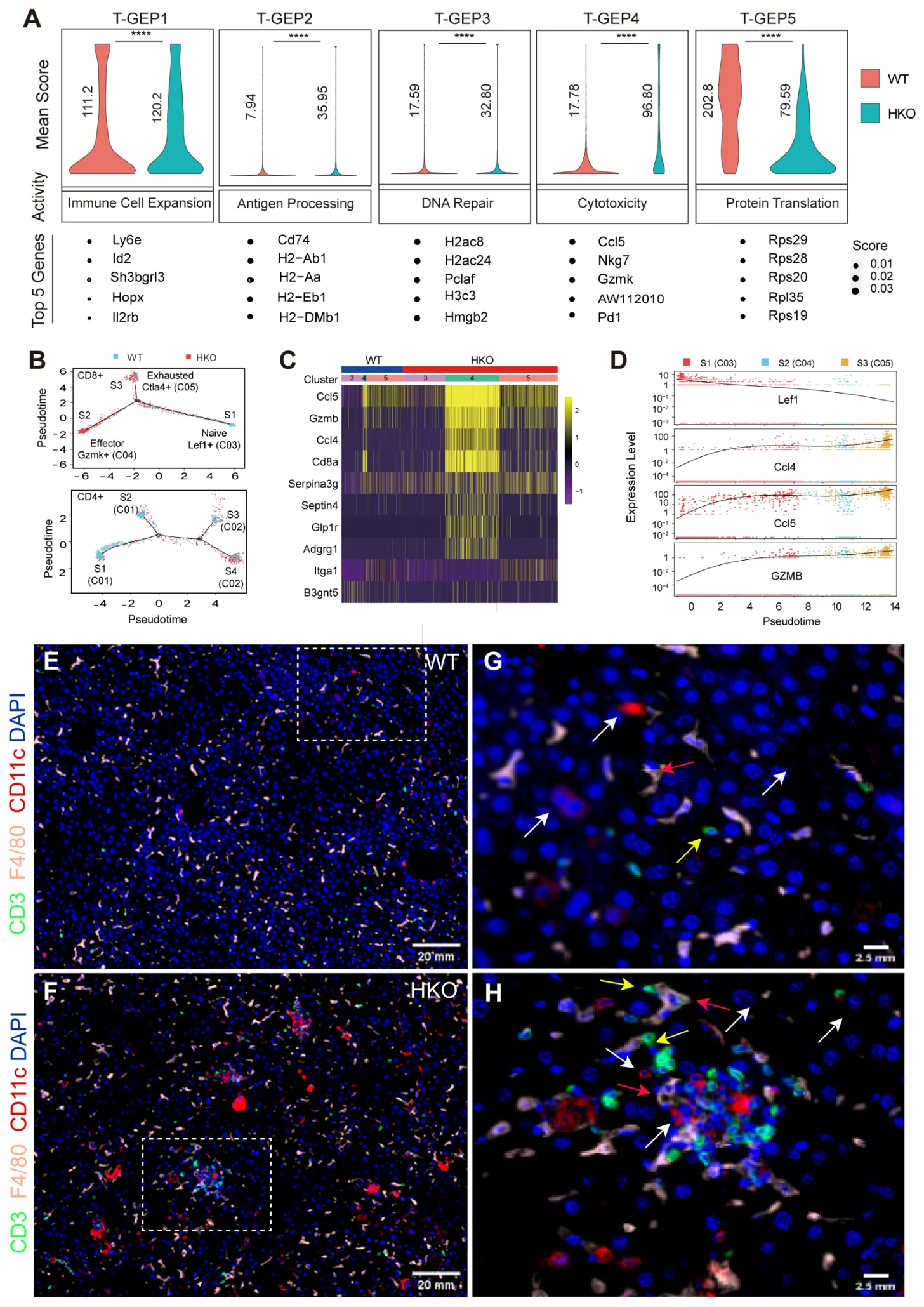
MASH-associated T lymphocytes show activated cell proliferation, DNA repair and effector function but repressed memory capacity. (**A**) Violin plots showing altered T cell gene expression programs (T-GEPs) in WT and HKO liver (numbers represent GEP activity scores, **** *p* < 0.0001 by two-tailed Student’s *t* test). GEP name and the top 5 genes are indicated underneath. (**B**) Trajectory charts showing developmental sequence of CD8+ (**upper**) and CD4+ (**lower**) clusters. Black solid lines represent the inferred trajectory. (**C**) Heatmap showing expression of the top 10 genes in the defined CD8+ T clusters. (**D**) Pseudotime charts showing expression of T cell marker genes. Black solid lines represent the inferred trajectory. (**E**,**F**) Higher-magnification views of the boxed regions in E and F, respectively. Immunofluorescent images showing co-localization of CD3 (green), CD11c (red) and F4/80 (pink) positive cells in WT and HKO liver tissues, respectively. Nuclei are DAPI-stained. (**G**,**H**) Higher-magnification views of the boxed regions in E and F, respectively. Yellow, red and white arrows indicate T cells, macrophages and dendritic cells, respectively.

Next, cNMF was performed to generate T cell-specific gene expression programs (T-GEPs). cNMF analysis yielded five distinct T-GEPs that are differentially activated between WT and HKO livers (Figure 4A, top panels). Functional examination of the top 30 genes of each T-GEP revealed that they were enriched for genes involved in immune cell expansion (T-GEP1), antigen processing (T-GEP2), DNA repair (T-GEP3), cytotoxicity (T-GEP4) and protein translation (T-GEP5), respectively (Figure 4A, middle panels). Notably, while T-GEP1 to T-GEP4 showed increased activities, T-GEP5, which regulates protein translation, showed decreased activity in HKO liver (Figure 4A, top panels). It is noteworthy that translational repression is a necessary process for inhibition of memory T cell formation [35]. These data suggest that hepatocyte ERAD deficiency is accompanied by the differentiation and expansion of effector T cells and the suppression of memory T cell formation.

Trajectory analysis was then performed using Monocle2 to determine the developmental origins for the defined CD8+ and CD4+ T cell clusters. C03, C04 and C05 represented CD8+ T cells at three distinct developmental states (states 1–3) (Figure 4B, top). The effector CD8+ T cell markers Ccl4, Ccl5, Gzmb and Septin4 were highly expressed in C04 (Figure 4C), whereas the naïve and exhausted CD8+ T cell markers, Lef1 and Ctla4, were expressed in C03 and C05, respectively (Figure 4B,C). Consistently, Lef1 showed a gradually decreasing expression, whereas Ctla4, Ccl4, Ccl5 and Gzmb showed gradually increasing temporal expression in the CD8+ T clusters (Figure 4D). These results suggest that clusters 03, 04 and 05 represent naïve, effector and exhausted CD8T cells, respectively. A similar developmental trajectory could also be seen for the CD4T clusters (Figure 4B, bottom), although no stage-specific markers were available to allow a definitive analysis. These results indicate that C01 and C02 might represent naïve and effector CD4+ T cells, respectively.

Lastly, immunofluorescence staining was performed to examine the spatial locations of T cells in relation to macrophages and dendritic cells in HKO livers. In both WT and HKO livers, macrophages and dendritic cells were widely and evenly distributed across sections (Figure 4E,F). Notably, T lymphocytes were frequently seen clustered together with macrophages and dendritic cells in HKO liver but not WT liver (Figure 4G,H). These findings support the notion that in MASH livers, antigen-laden macrophages and dendritic cells come in close contact with and prime naïve T cells, which proliferate and differentiate locally to become “DAMP-clearing” effector T cells.

### 3.5. Immunological Depletion of CD8+ T Cells Aggravates ERAD Deficiency-Induced Hepatic Inflammation and Liver Injury

**Figure 5:**
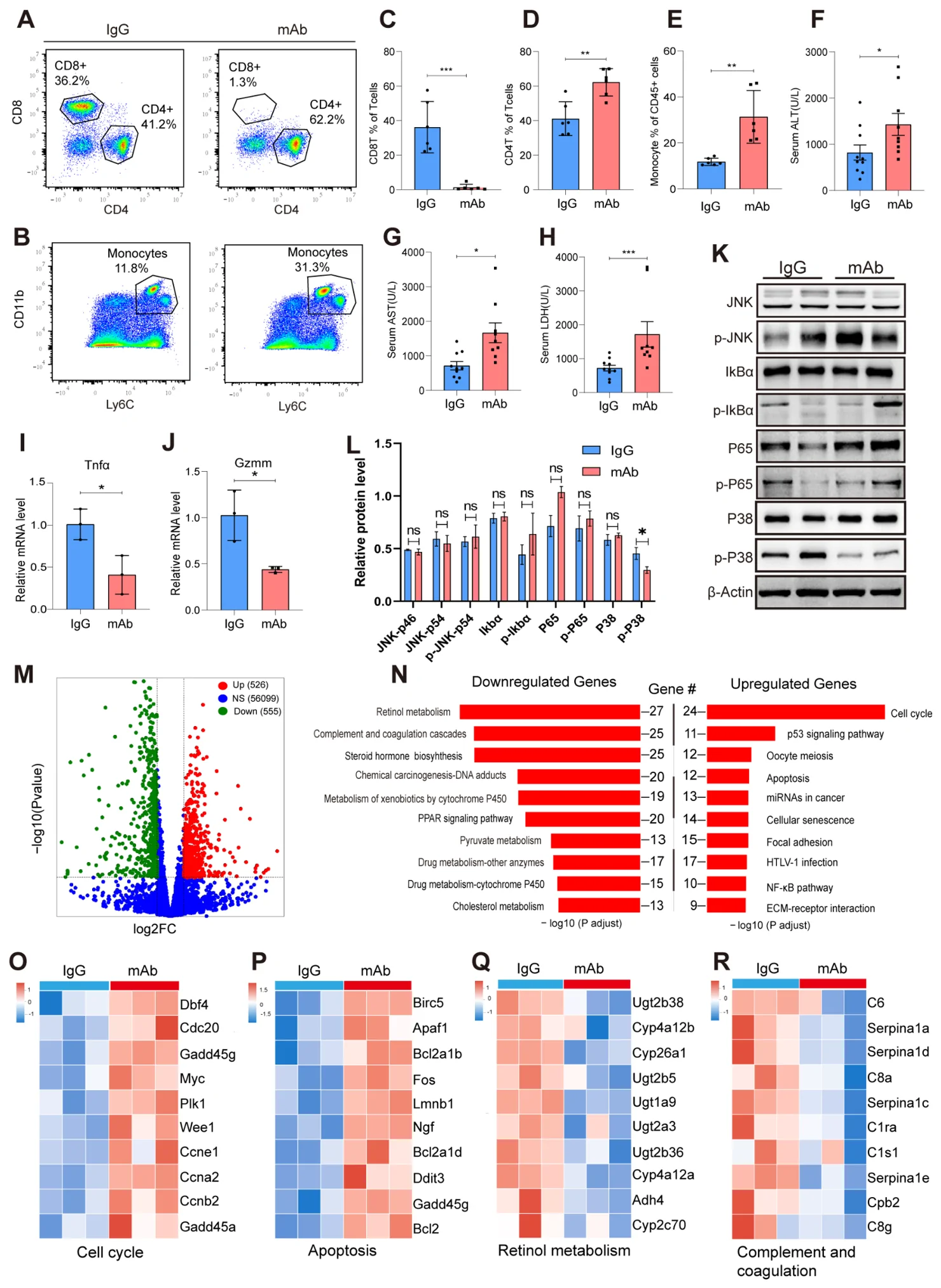
Immunological depletion of CD8+ T cells aggravates ERAD deficiency-induced hepatocyte inflammation and injury. (**A**,**B**) Flow cytometry gating for quantification of CD4+ and CD8+ T lymphocytes (**A**) and monocytes (**B**) in IgG (*n* = 6) and Cd8α mAb (*n* = 6) administered mice. (**C**–**E**) Quantification of CD8+ T (**C**), CD4+ T (**D**) and monocytes (**E**). (**F**–**H**) Serum ALT (**F**), AST (**G**) and LDH (**H**) (*n* = 10 or 9 per group). (**I**,**J**) qRT-PCR analysis of proinflammatory genes. (**K**,**L**) Representative immunoblots (**K**) and corresponding quantification (**L**) of stress signaling molecules. (**M**,**N**) Scatter plot (**M**) and KEGG pathway chart (**N**) showing differentially expressed genes and enriched pathways (*n* = 3 per group). (**O**–**R**) Heatmaps showing representative up- (**O**,**P**) and down- (**Q**,**R**) regulated genes in four different pathways (*n* = 3 per group). All data are presented as means ± SEM. ns, not significant; * *p* < 0.05, ** *p* < 0.01, *** *p* < 0.001, by two-tailed Student’s *t* test.

To understand the roles of self-antigen-primed CD8+ T cells during MASH pathogenesis, we intraperitoneally injected a CD8α-neutralizing monoclonal antibody (CD8α mAb) to deplete CD8+ T lymphocytes in HKO mice. Flow cytometry analysis revealed that CD8α mAb administration did not significantly alter the hepatic frequencies of total immune cells (CD45+ cells) (Figure S6A,B), macrophages (Figure S6C,D), Kupffer cells and myeloid-derived macrophages (Figure S6E–G). However, CD8α mAb caused a near 30-fold reduction in hepatic frequency of CD8+ T cells, as compared to that in control IgG-injected mice (Figure 5A,C). Intriguingly, frequencies of CD4+ T cells (Figure 5A,D) and monocytes (Figure 5B,E) were markedly increased by CD8α mAb delivery. These observations suggest that the CD8α-neutralizing antibody used in this study has high efficacy in specifically depleting CD8+ T cells in mice, as has been demonstrated in numerous previous studies [36,37].

CD8α mAb-treated HKO mice showed body weight gain and liver/body weight ratio comparable to those of control (IgG-treated) mice (Figure S6H,I). However, these mice had significantly higher levels of serum ALT, AST and LDH (Figure 5F–H). Furthermore, histological analysis revealed markedly more Councilman bodies (Figure S6N–R). These observations indicate that depletion of CD8+ T cells exacerbates ERAD deficiency-induced hepatocyte apoptosis. To assess the functional impact of CD8+ T depletion on hepatic inflammation, quantitative RT-PCR was first performed to analyze the mRNA expression of several proinflammatory genes. While IL-1b, IL6, Gzma and Gzmb expression remained unchanged, TNFα and Gzmm expression were significantly reduced in CD8α mAb-treated livers (Figure S6J–M, Figure 5I,J). Both TNFα and Gzmm are actively transcribed in CD8+ T cells. The reduced hepatic levels of TNFα and Gzmm mRNAs after CD8+ T cell depletion are likely due to the direct loss of CD8+ T-cell-derived transcripts, rather than an indication of reduced hepatic inflammation. In fact, the substantial accumulation of monocytes, together with the fact that serum ALT, AST and LDH levels and the number of hepatic Councilman bodies were increased, strongly indicates that CD8+ T cell depletion exacerbates hepatic inflammation and injury. Next, immunoblotting was used to assess the hepatic expression of several inflammatory and stress-responsive kinases and their phosphorylated forms following CD8α depletion. CD8α depletion did not impact JNK, IkBα and P65 expression but markedly decreased the expression of phosphorylated P38 (Figure 5K,L).

To more comprehensively assess the impact of CD8α depletion on hepatic function and stress signaling, we profiled the hepatic transcriptome of IgG- and CD8α mAb-treated mice. A total of 1081 differentially expressed genes (DEGs) were identified, including 526 up- and 555 downregulated genes (Figure 5M). Of note, Kyoto Encyclopedia of Genes and Genomes (KEGG) pathway enrichment analysis showed that most upregulated genes participated in cell cycle regulation, cellular senescence, and cell death signaling (Figure 5N(right),O,P), whereas the downregulated genes were mainly involved in nutrient metabolism and complement function (Figure 5N(left),Q,R). Collectively, these results support the hypothesis that hepatocyte antigen-primed CD8+ T cells overall play a protective role in the liver during pathogenesis of MASH, likely by limiting hepatic inflammation and accelerating clearance of damaged hepatocytes.

### 3.6. Hepatocyte ER-Stress Gene Expression Correlates with Myeloid Enrichment in Human MASH Livers

**Figure 6:**
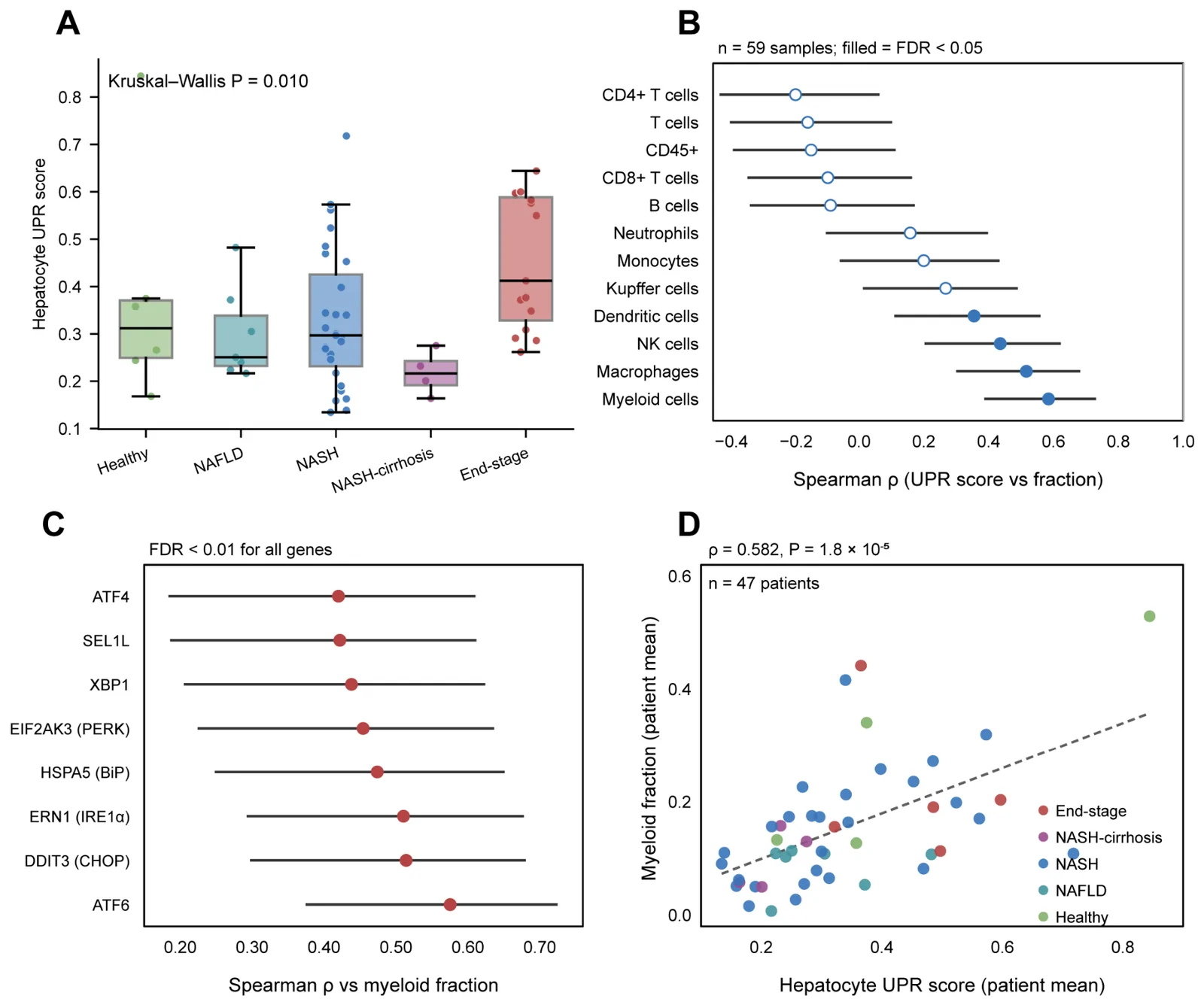
Hepatocyte UPR scores and immune-cell correlations in human MASH livers. (**A**) Hepatocyte UPR scores by diagnostic group (healthy, *n* = 6; NAFLD, *n* = 7; NASH, *n* = 27; NASH with cirrhosis, *n* = 4; end-stage disease, *n* = 15). Boxes show the median and interquartile range; whiskers extend to 1.5 × IQR; dots represent individual samples. *p* value was calculated with the Kruskal–Wallis test. (**B**) Spearman correlation coefficients (ρ) with 95% confidence intervals (Fisher z-transformation) between hepatocyte UPR scores and the fraction of each immune population (*n* = 59 samples). Each point represents one immune population. Filled circles denote FDR < 0.05 and open circles denote FDR ≥ 0.05 after Benjamini–Hochberg correction across the 12 populations. (**C**) Spearman correlation coefficients (with 95% confidence intervals) between the hepatocyte expression of each of eight core UPR/ERAD genes (HSPA5, ERN1, XBP1, DDIT3, ATF4, EIF2AK3, ATF6, SEL1L) and the myeloid cell fraction (*n* = 59 samples); Each red point represents one gene; FDR < 0.01 for all eight genes. (**D**) Patient-level correlation between hepatocyte UPR scores and the myeloid cell fraction (*n* = 47 patients; each point is the mean across samples from one patient). The dashed line shows linear regression.

To assess whether this relationship is conserved in human MAFLD/MASH, we re-analyzed a published liver snRNA-seq cohort (GSE202379) containing 47 MAFLD/MASH patients [28]. Hepatocytes were identified by ALB expression, while a composite UPR score was derived from eight UPR/ERAD genes (Section 2.14). UPR scores differed across diagnostic groups, with a modest increase for the mid-stage group (Spearman ρ = 0.269, *p* = 0.040), and the highest increase in the end-stage group (Kruskal–Wallis *p* = 0.0096; Figure 6A). UPR scores also showed a positive association with immune-cell abundances (Figure 6B). Specifically, hepatocyte UPR scores correlated positively with the abundance of total myeloid cells (Spearman ρ = 0.585, *p* = 1.2 × 10^−6^, *n* = 59), macrophages (ρ = 0.516, *p* = 2.9 × 10^−5^), NK cells (ρ = 0.436, *p* = 5.6 × 10^−4^) and dendritic cells (ρ = 0.355, *p* = 5.8 × 10^−3^). However, not all immune populations followed this pattern. UPR scores showed no correlation with the abundance of Kupffer cells, CD8+ T and total CD45+ cells. Among the examined UPR markers, ATF6 (ρ = 0.575), DDIT3 (CHOP; ρ = 0.514) and ERN1 (IRE1α; ρ = 0.510) showed the strongest correlations with myeloid abundance (all FDR < 0.001; Figure 6C). These associations persisted at the patient level (ρ = 0.582, *p* = 1.8 × 10^−5^, *n* = 47; Figure 6D). Together, these findings provide evidence that hepatocyte ER stress is associated with remodeling of hepatic myeloid landscape during human MAFLD/MASH progression.

## 4. Discussion

Dysregulated immune activation promotes hepatocyte injury and contributes substantially to the pathogenesis of Metabolic Dysfunction-Associated Steatohepatitis (MASH). To define the lineage-specific functions and intercellular interactions of hepatic immune cells during MASH development, we combined single-cell RNA sequencing (scRNA-seq) with flow cytometry to characterize the hepatic immune landscape in a novel spontaneous mouse model of MASH. Unlike most diet-induced models, in which disease development is driven by excess or deficiency of multiple nutrients, the model used in this study was generated through hepatocyte-specific disruption of endoplasmic reticulum-associated degradation (ERAD). Mice with ERAD deficiency exhibit robust hepatic immune activation as early as three months of age, before the development of overt pathological abnormalities such as substantial lipid accumulation, fibrosis, or hepatocellular carcinoma. This genetically defined model therefore provides a relatively controlled background in which to investigate early immune events associated with MASH progression.

Hepatocyte ERAD deficiency markedly expanded multiple hepatic immune populations, including monocytes, macrophages, dendritic cells (DCs), and CD8+ T lymphocytes. Expanded myeloid cells exhibited enhanced transcriptional programs associated with phagocytosis, antigen processing and presentation, whereas T lymphocytes displayed increased proliferative and effector signatures. Co-immunostaining further revealed close spatial associations between myeloid cells and CD8+ T cells within ERAD-deficient livers, suggesting active communication between the innate and adaptive immune compartments. Importantly, antibody-mediated depletion of CD8+ T cells exacerbated hepatic inflammation and injury. Collectively, these findings reveal a coordinated innate–adaptive immune response to hepatocyte metabolic stress and identify an unexpected protective role for CD8+ T cells in limiting inflammation and tissue damage during MASH progression.

Our scRNA-seq analysis revealed no significant activation of ER stress-related genes in hepatic immune cells from HKO livers. This finding indicates that the immune expansion and functional reprogramming observed in HKO livers are unlikely to be driven by immune cell-intrinsic ER stress, but instead by signaling cues from ER-stressed or damaged hepatocytes. Mechanistically, it has been shown that ER stress promotes the release of damage-associated molecular patterns and alters hepatocyte secretory profiles [2,3].

Notably, by analyzing a publicly available liver snRNA-seq dataset of 47 MAFLD/MASH patients (GSE202379) [28], we found a positive correlation between hepatocyte UPR scores and abundances of hepatic myeloid cells (ρ = 0.585, *p* = 1.2 × 10^−6^) and macrophages (ρ = 0.516, *p* = 2.9 × 10^−5^). Both associations persisted at the patient level (*n* = 47; ρ = 0.582, *p* = 1.8 × 10^−5^). Among the examined UPR markers, ATF6, DDIT3 (CHOP), and ERN1 (IRE1α) showed the strongest correlation with myeloid abundance. Thus, the hepatocyte ER-stress–myeloid axis defined in our ERAD-deficient model appears to operate in human MASH, underscoring the relevance of our HKO model to human MASH.

Consistent with previous reports, hepatic stellate cells and endothelial cells emerged as major signaling hubs in HKO livers. Among the upregulated ligand–receptor pairs, including MDK, SEMA4D/SEMA4C–PLXNB1/2, NOTCH2–JAG1 and FGF/BDNF signaling, several can regulate myeloid-cell migration, activation and survival. For example, both MDK and SEMA4D–PLXNB2 signaling have been implicated in the recruitment of monocytes and macrophages [38,39,40]. The concurrent expansion of macrophages, DCs and T cells in these livers likely results from a rewired intercellular network that favors immune-cell recruitment and activation.

Innate immune cells constitute the first line of defense against both pathogens and sterile tissue injury [41]. Hepatocyte ERAD deficiency induced extensive remodeling of the hepatic innate immune compartment, characterized by the expansion of several myeloid populations, including macrophages, monocytes, DCs, and granulocytes. Although the number of resident Kupffer cells (KCs) decreased, myeloid-derived macrophages significantly increased, resulting in an overall expansion of the hepatic macrophage compartment. These changes are consistent with those reported in diet-induced models of MASH, in which resident KCs are progressively depleted or functionally impaired and are replaced by recruited myeloid-derived macrophages during disease progression [42].

In addition to their phagocytic functions, hepatic myeloid cells contribute to antigen processing and presentation and thereby connect innate sensing of tissue damage with adaptive immune activation [5]. To quantitatively assess functional changes in hepatic myeloid cells following hepatocyte ERAD deficiency, we applied consensus non-negative matrix factorization (cNMF) to identify and score distinct gene expression programs. Two myeloid programs associated with antigen processing and presentation were markedly enriched in ERAD-deficient livers. Together with the increased expression of phagocytosis-related genes, these findings indicate that hepatocyte ERAD deficiency enhances the clearance capacity and antigen-presenting functions of hepatic myeloid cells.

A macrophage population characterized by high Trem2 expression was selectively enriched in ERAD-deficient livers. These Trem2-high macrophages expressed molecular markers previously associated with lipid-associated macrophages, including Gpnmb, Mmp12 and Cd63 (Figure 3D) [43]. Although Trem2^+^ macrophages and lipid-associated macrophages have been described in MASH [44,45], the functional heterogeneity among Trem2-expressing macrophages remains incompletely understood. To further resolve their functional states, we stratified the KC population into Trem2-high and Trem2-low subsets according to their transcriptomic profiles. Relative to Trem2-low KCs, Trem2-high KCs showed increased expression of genes involved in phagocytosis, lipid metabolism, lysosomal activity and antigen processing and presentation (Figure S4B,C), indicating a broader functional repertoire. These findings suggest that Trem2-high KCs may integrate lipid handling and phagocytic clearance with antigen presentation during MASH progression. Notably, analogous Trem2-high macrophage populations have been identified in liver biopsies from patients with MASH and in other rodent models of the disease [15,46].

To explore the intracellular location and potential subcellular functions of TREM2, we initially attempted to generate THP-1 cells stably expressing TREM2-GFP. However, THP-1 cells were highly sensitive to transfection and selection stress, undergoing rapid differentiation and cell death, which prevented the establishment of stable lines. We therefore used HepG2 cells as a complementary experimental system because of their greater tolerance to transfection and antibiotic selection. Although HepG2 cells do not reproduce the cellular context of hepatic macrophages, this approach enabled us to examine the intracellular distribution of TREM2. The observed lysosomal localization of TREM2 (Figure 3E–R) provides preliminary evidence that TREM2 may participate in intracellular trafficking or lysosome-associated clearance pathways. Nevertheless, these observations should be considered hypothesis-generating and require validation in macrophage-relevant models. Collectively, our data suggest that Trem2-high macrophages represent an activated hepatic macrophage state characterized by enhanced lipid handling, lysosomal clearance and antigen processing capacity in ERAD-deficient livers. However, whether TREM2 directly regulates these functions remains unresolved. Studies using myeloid-specific Trem2 deletion or other macrophage-targeted genetic approaches will be required to establish the causal role of TREM2 in hepatic macrophage function and MASH progression.

Although clinical and experimental evidence has implicated adaptive immunity in MASH pathogenesis [47], the responses of individual adaptive immune cell populations to metabolically stressed or damaged hepatocytes remain insufficiently defined. In the present study, hepatocyte ERAD deficiency was associated with an expansion of hepatic T lymphocytes and a reduction in B lymphocytes. The expanded T-cell compartment included CD4+, CD8+, and CD4-CD8-double-negative populations. Furthermore, developmental trajectory analysis suggested that the expanded effector C04 and exhausted C05 CD8+ T cell clusters may arise from the naive C03 population. These findings support a model in which metabolically stressed hepatocytes initiate immune signals that promote the recruitment, activation, expansion and differentiation of naïve CD8+ T cells within the liver.

The functional role of these putatively self-antigen-primed hepatic CD8+ T cells during MASH progression remains unclear. To address this question experimentally, we depleted CD8+ T cells in mice with hepatocyte ERAD deficiency. Unexpectedly, depletion of circulating CD8+ T cells exacerbated, rather than ameliorated, hepatic inflammation and injury. This result indicates that the expanded CD8+ T-cell population in ERAD-deficient mice is not uniformly pathogenic and may instead exert a net hepatoprotective effect under these conditions.

The mechanisms underlying CD8+ T-cell-mediated hepatoprotection remain to be determined. Notably, CD8+ T-cell depletion markedly increased hepatic monocyte accumulation in ERAD-deficient mice, raising the possibility that CD8+ T cells are directly or indirectly involved in regulating the recruitment, survival, or local proliferation of monocytes. Of note, this process occurs without affecting the hepatic MDM population. Although further studies are needed to define the molecular basis underlying this finding, one can speculate that CD8+ T cells may be required for monocyte-to-macrophage differentiation or the ongoing turnover within the macrophage compartment.

The identity of the protective CD8+ T cell population in ERAD-deficient livers remains to be resolved. Two functional CD8+ T subsets are worth mentioning: the effector CD8+ T subset and the memory CD8+ T subset. Both subsets have the capacity to limit injury by clearing damaged hepatocytes, reducing DAMP-driven monocyte recruitment and providing protection against liver-stage infection [48,49]. However, our scRNA-seq data showed that while the effector CD8+ T subset was expanded, the memory CD8+ T program was inhibited in ERAD-deficient livers. Therefore, it is conceivable that effector, not memory, CD8+ T cells are responsible for the demonstrated protective effects in ERAD-deficient livers. Defining the clonality, antigen specificity, spatial distribution, and molecular characteristics of the protective CD8+ T subset will be important for understanding their contribution to liver homeostasis and for designing strategies that preserve hepatoprotective states while avoiding tissue-damaging responses.

Our findings further emphasize the context-dependent functions of CD8+ T cells in metabolic liver disease. Although activated or exhausted CD8+ T cells have frequently been associated with hepatocyte injury and MASH progression, distinct CD8+ T-cell subsets may perform divergent or even opposing functions depending on disease stage, antigen specificity, differentiation state, and the surrounding cytokine and metabolic environment. The protective effect observed in the ERAD-deficient model may therefore reflect the activity of a specific CD8+ T-cell population that regulates inflammatory myeloid-cell responses or facilitates the controlled clearance of damaged cells. Defining the clonality, antigen specificity, spatial distribution, and molecular characteristics of these cells will be important for understanding their contribution to liver homeostasis.

Several limitations of this study should be acknowledged. First, only male mice were used. Sex-dependent differences in metabolism, immune cell composition, and MASH susceptibility may influence the hepatic immune landscape and the protective function of CD8+ T cells. Future studies incorporating both male and female animals are therefore required to determine whether observed immune responses are sex dependent. Second, the intracellular location of TREM2 was examined in HepG2 cells rather than in macrophages. Although this system provided initial information regarding TREM2 trafficking, the findings require confirmation in primary hepatic macrophages, macrophage cell lines, or myeloid-specific genetic models. Third, antibody-mediated CD8+ T-cell depletion does not identify the specific CD8+ T-cell subset or effector mechanism responsible for hepatoprotection. More selective genetic and functional approaches will be needed to resolve the contributions of individual CD8+ T-cell states. Fourth, our human validation is cross-sectional in design. The association between hepatocyte ER-stress gene expression and myeloid abundance is correlative rather than causal. Immune populations were defined by marker expression, and TREM2+ macrophages and CD8+ T-cell subsets were poorly represented in nuclear transcriptomes. Spatially resolved or immune-cell-enriched datasets will be required to test subset specificity and the direction of the effect.

In summary, this study provides a single-cell characterization of the hepatic immune landscape in hepatocyte ERAD deficiency-driven MASH and reveals extensive coordination between innate–adaptive immune compartments in response to hepatocyte stress. Hepatocyte ERAD deficiency promoted the expansion and functional activation of multiple myeloid populations, including a Trem2-high macrophage state associated with lipid handling, lysosomal clearance, and antigen presentation. At the same time, hepatic CD8+ T cells underwent expansion and differentiation and displayed an unexpected protective function, as their depletion increased monocyte accumulation, hepatic inflammation and tissue injury. These findings broaden our understanding of immune-cell heterogeneity in MASH and demonstrate that CD8+ T-cell responses are not necessarily pathogenic. Therapeutic approaches that selectively preserve or enhance hepatoprotective CD8+ T-cell states, while avoiding the activation of tissue-damaging subsets, warrant further investigation as potential strategies for the treatment of MASH.

## Figures and Tables

**Table 1:** Experimental model and study participant details.

Experimental Models: Cell Lines	Source	Identifier
Human: HepG2	In house	N/A
Experimental models: Organisms/strains	Source	Identifier
Mouse: Sel1l^F/F^ on C57BL/6 background (male)	In house	N/A
Mouse: Sel1l^F/F^; Alb-Cre on C57BL/6 background (male)	In house	N/A

**Table 2:** Antibodies.

Flow Cytometry Antibodies	Source	Identifier
Anti-mouse CD16/CD32 (2.4G2)	BD Pharmingen (San Diego, CA, USA)	Cat#553141
Anti-CD45 (30-F11)	Biolegend (San Diego, CA, USA)	Cat#103107
Anti-CD3 (17A2)	Biolegend	Cat#100217
Anti-CD4 (RM4-5)	Biolegend	Cat#100515
Anti-CD8a (53-6.7)	Biolegend	Cat#100751
Anti-CD11b (M1/70)	Biolegend	Cat#101215
Anti-F4/80 (BM8)	Biolegend	Cat#123131
Anti-Ly6C (HK1.4)	Biolegend	Cat#128007
Anti-CD45 (30-F11)	eBioscience	Cat#48-0451-82
Anti-CD3 (17A2)	eBioscience	Cat#69-0032-82
Anti-CD4 (RM4-5)	eBioscience	Cat#45-0042-82
Anti-CD8a (53-6.7)	eBioscience	Cat#45-0081-82
Anti-CD11b (M1/70)	eBioscience	Cat#11-0112-82
Anti-F4/80 (BM8)	eBioscience	Cat#17-4801-82
Anti-Gr1 (RB6-8C5)	eBioscience	Cat#17-5931-82
Anti-CD19 (1D3)	eBioscience	Cat#25-0193-82
Anti-CD11c (N418)	eBioscience	Cat#25-0114-82
Anti-MHCII (AF6-120.1)	eBioscience	Cat#12-5320-82
Anti-NK1.1 (PK136)	eBioscience	Cat#12-5941-82
IHC antibodies	Source	Identifier
Anti-F4/80 (D2S9R)	CST (Danvers, MA, USA)	Cat#70076
Anti-α-Smooth	CST	Cat#19245
Anti-Rabbit HRP conjugate	Jackson Immuno Research (West Grove, PA, USA)	Cat#111-035-045
Mouse I.P. antibodies	Source	Identifier
Anti-mouse CD8a (2.43)	Bio X Cell (Lebanon, NH, USA)	Cat#BE0061
Anti-IgG2 isotype (LTF-2)	Bio X Cell	Cat#BE0090
Chemicals, Medium and Kit	Source	Identifier
Fixable viability Dye eFluor780	eBioscience	Cat#69-0865-14
Collagenase IV	Sigma (St. Louis, MO, USA)	Cat#C5138
Collagen I	Sigma	Cat#C3867
Mito Tracker	Thermo Fisher (Waltham, MA, USA)	Cat#M22426
Lyso Tracker	Thermo Fisher	Cat#L12492
Hygromycin B	Thermo Fisher	Cat#10687010
Doxycycline hydrochloride	Sangon Biotech (Shanghai, China)	Cat#A600889

**Table 3:** Gene-specific PCR primers.

Primers	Forward	Reverse
Tnfα	CAGGCGGTGCCTATGTCTC	CGATCACCCCGAAGTTCAGTAG
Gzmm	GAGAGAGTGGGGAACCCTTTC	CTCTCGACCCCCAATGATCT
Il1β	GAAATGCCACCTTTTGACAGTG	TGGATGCTCTCATCAGGACAG
Il6	TCTATACCACTTCACAAGTCGGA	GAATTGCCATTGCACAACTCTTT
Gzma	TGCTGCCCACTGTAACGTG	GGTAGGTGAAGGATAGCCACAT
Gzmb	CCTGCTACTGCTGACCTTGT	GGGATGACTTGCTGGGTCTT

## Data Availability

The datasets generated and/or analyzed during the current study are available in the Genome Sequence Archive (GSA) repository at the National Genomics Data Center (Beijing Institute of Genomics, Chinese Academy of Sciences/China National Center for Bioinformation), under accession numbers CRA039675 and CRA039846, and are publicly accessible at https://ngdc.cncb.ac.cn/gsa/browse/CRA039675 (accessed on 23 August 2026) and https://ngdc.cncb.ac.cn/gsa/browse/CRA039846 (accessed on 23 August 2026).

## Supplementary Materials

The following supporting information can be downloaded at: https://www.mdpi.com/article/10.3390/cells15171562/s1. Figure S1: Phenotype of hepatocyte-specific ERAD-deficient mice; Figure S2: Hepatocyte-specific ERAD deficiency does not alter total T, CD4T, DC, B and granulocyte populations in the liver; Figure S3: Hepatocyte-specific ERAD deficiency induces changes in myeloid cell sub-types; Figure S4: Trem2-high-expressing KCs populations are highly activated in antigen processing and presentation; Figure S5: Hepatocyte-specific ERAD deficiency induces changes in T lymphocyte sub-types; Figure S6: Short-term administration of CD8α mAb does not alter the abundance of total hepatic immune cells and macrophages; Figure S7: Flow cytometry gating strategy. Table S1: Marker genes used for T cell subset identification.

## Abbreviations

The following abbreviations are used in this manuscript:

ERAD	Endoplasmic reticulum-associated degradation
MASH	Metabolic Dysfunction-Associated Steatohepatitis
MAFLD	Metabolic Dysfunction-Associated Fatty Liver Disease
DAMPs	Damage-associated Molecular Patterns
scRNA-seq	Single-cell RNA sequencing
cNMF	Consensus non-negative matrix factorization
GEPs	Gene expression programs
M-GEPs	Myeloid-specific gene expression programs
T-GEPs	T cell-specific gene expression programs
MDMs	Myeloid-derived macrophages
KCs	Kupffer cells
Trem2	Triggering receptor expressed on myeloid cells 2
DNT	Double negative T
KEGG	Kyoto Encyclopedia of Genes and Genomes
UPR	Unfolded protein response
