# Supplementary material for: Hepatocyte-Specific Deficiency of Endoplasmic Reticulum-Associated Degradation Induces Coordinated Innate–Adaptive Immune Responses
Source: Cells. 2026 Aug 28;15(17):1562. doi: 10.3390/cells15171562 (PMC13564904; doi:10.3390/cells15171562)
Supplement: Supplementary file 1 [file cells-15-01562-s001.zip › cells-4469884-supplementary.pdf]

Figure S1. Phenotype of hepatocyte-specific ERAD-deficient mice. (A-D) Serum ALT, AST, TBIL and LDH levels in 3-month-old WT vs HKO mice (*n* = 6 per group). (E-G) Protein and mRNA expression in liver tissues from 3-month-old WT and HKO mice (*n* = 4 per group). (E) Western blot analysis of IRE1α, PERK and p-PERK; the dashed box highlights the p-PERK bands. (F) qPCR analysis of ATF6 mRNA expression. (G) qPCR analysis of CHOP mRNA expression. (H) Transmission electron microscopy of primary hepatocytes from 3-month-old mice. Black arrows: mitochondria; red arrows: endoplasmic reticulum. (I) Liver tissue section from a 6-month-old mouse. Black arrows: hepatocyte nuclei; red arrows: granulocytes. (J) Oil Red O staining of liver tissue from a 6-month-old mouse. Arrows indicate lipid droplets. Data in A-D are means ± SEM. ns, not significant; $*p < 0.05$, $**p < 0.01$, $***p < 0.001$, $****p < 0.0001$; two-tailed Student's t-test.

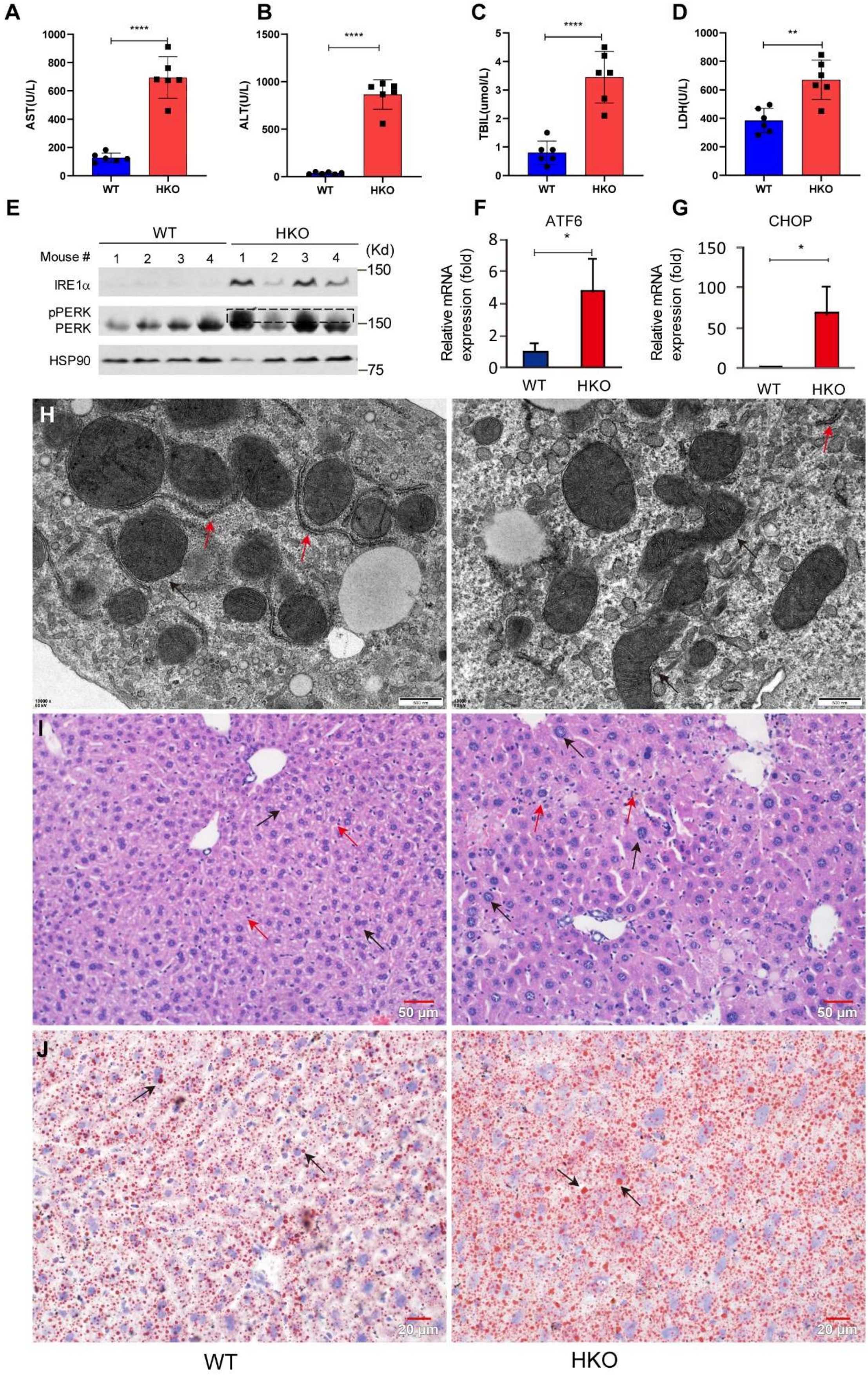

A
AST(U/L)
1000
800
600
400
200
0
****
WT
HKO
B
ALT(U/L)
1500
1000
500
0
****
WT
HKO
C
TBIL(umol/L)
5
4
3
2
1
0
****
WT
HKO
D
LDH(U/L)
1000
800
600
400
200
0
**
WT
HKO
E
WT
HKO
(Kd)
Mouse #
1
2
3
4
1
2
3
4
IRE1α
–150
pPERK
PERK
–150
HSP90
–75
F
ATF6
Relative mRNA expression (fold)
8
6
4
2
0
*
WT
HKO
G
CHOP
Relative mRNA expression (fold)
150
100
50
0
*
WT
HKO
H
I
50 µm
50 µm
J
20 µm
20 µm
WT
HKO

Figure S2 Hepatocyte-specific ERAD deficiency does not alter total T, CD4T, DC, B and granulocyte populations in the liver. (A-E) FACS gating strategies for monitoring total T cells (A), CD4+ T cells (B), DCs (C), MHCII+ DCs (D), B cells (E). (F-K) Quantification of total T cells (F), CD4+ T cells (G), DCs (H), MHCII+ DCs (I), B cells (J) and granulocytes (K) (n = 6 per genotype). All data are presented as means ± SEM (n = 6). ns, not significant; *$p < 0.05$, **$p < 0.01$, ***$p < 0.001$, ****$p < 0.0001$ by two-tailed Student's t test.

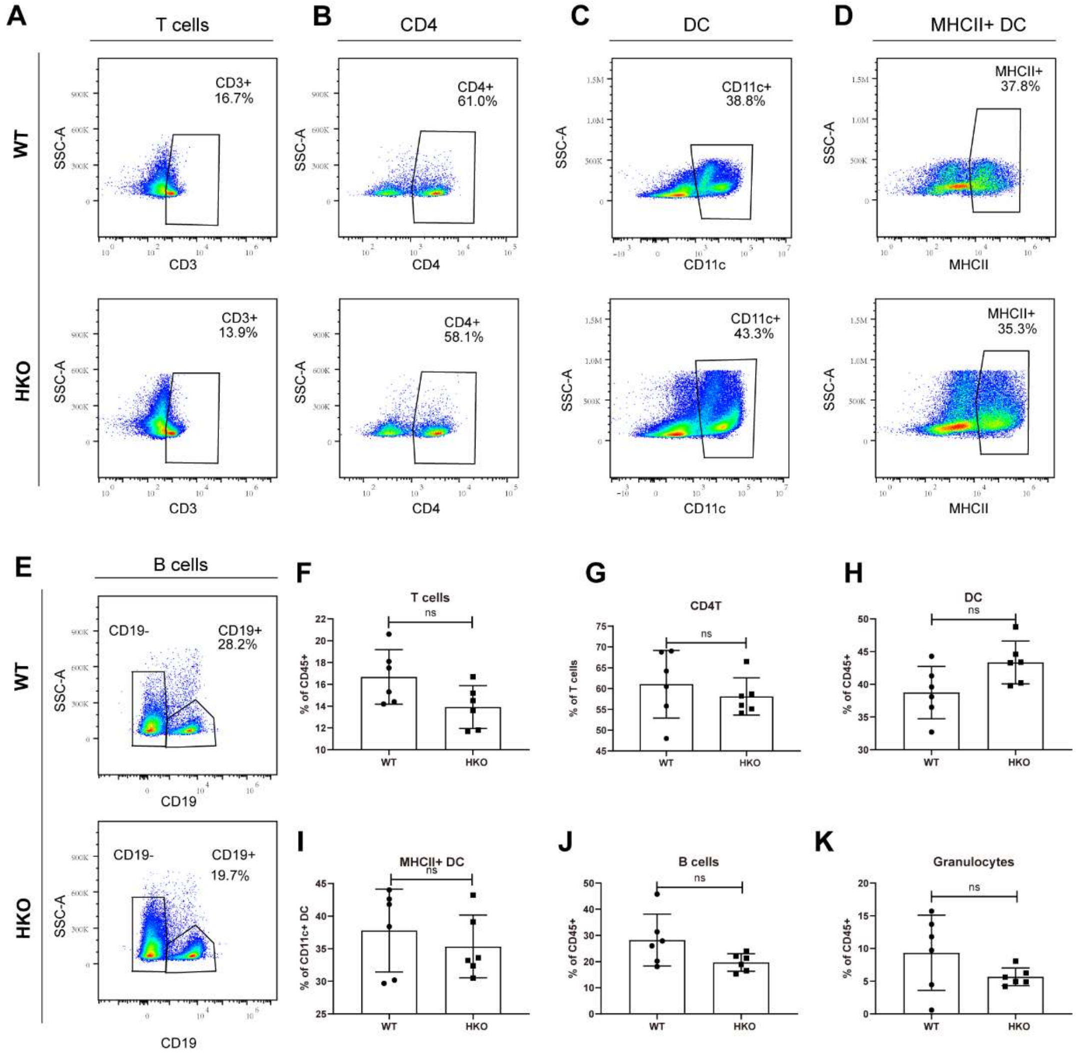

Figure S3 Hepatocyte-specific ERAD deficiency induces changes in myeloid cell sub-types. (A-C) t-SNE plots based on separated WT (A) and HKO (B) and integrated (C) scRNA-seq data showing the defined myeloid cell subtypes. (D) Stacked bar chart showing relative abundances of the defined myeloid cell subtypes in WT vs HKO liver.

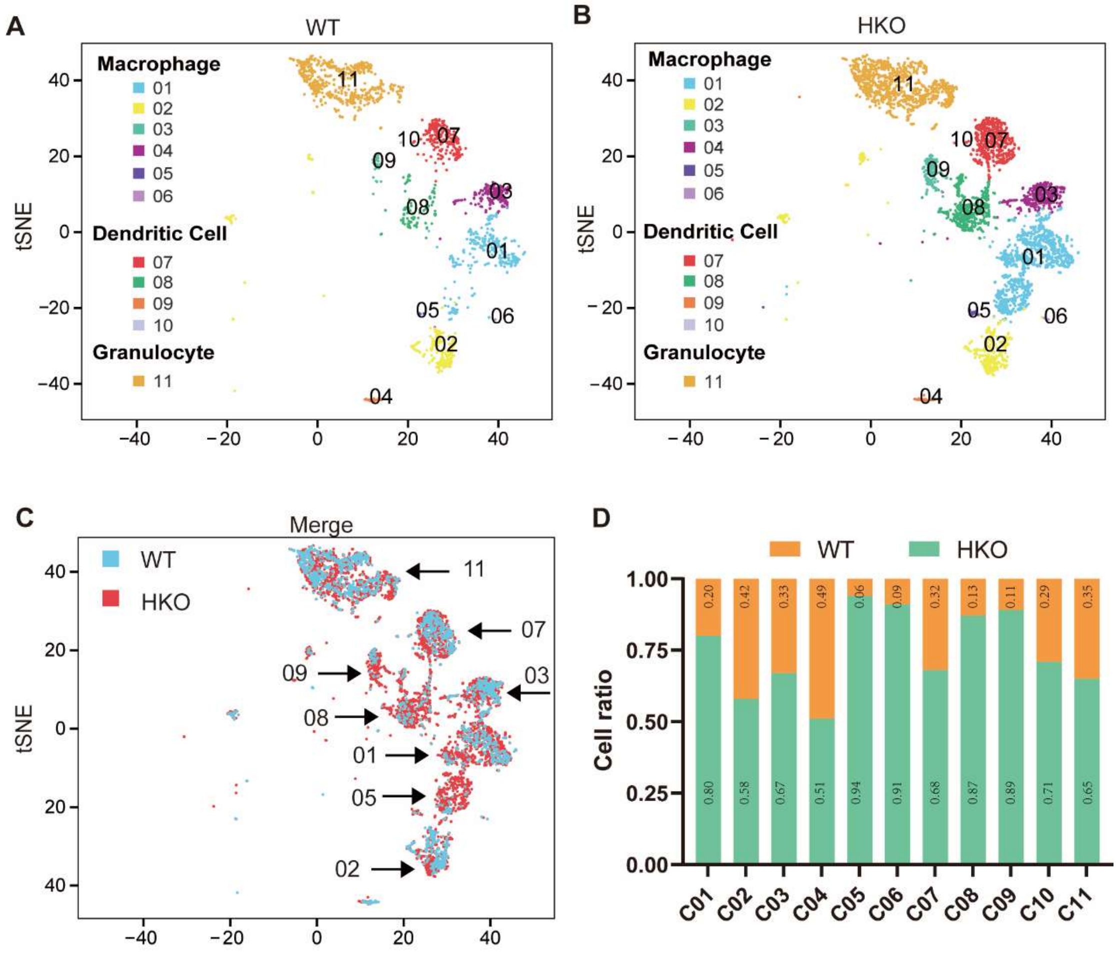

Figure S4 The Trem2-high-expressing KC populations are highly activated in antigen processing and presentation. (A) t-SNE plots showing spatial expression of M1 and M2 type macrophage markers in Trem2-low and Trem2-high myeloid clusters. Each dot represents a single cell, and each dot is colored according to the transcript count. (B-C) Bar charts indicating enriched KEGG pathways in Trem2-low and Trem2-high expressing cells. (D) t-SNE plots showing the spatial distribution of MDM, Trem2-low, and Trem2-high-expressing cells in both WT and HKO livers. (E) Heatmap showing antigen presentation-related genes expression levels in Trem2-low versus Trem2-high KC cells.

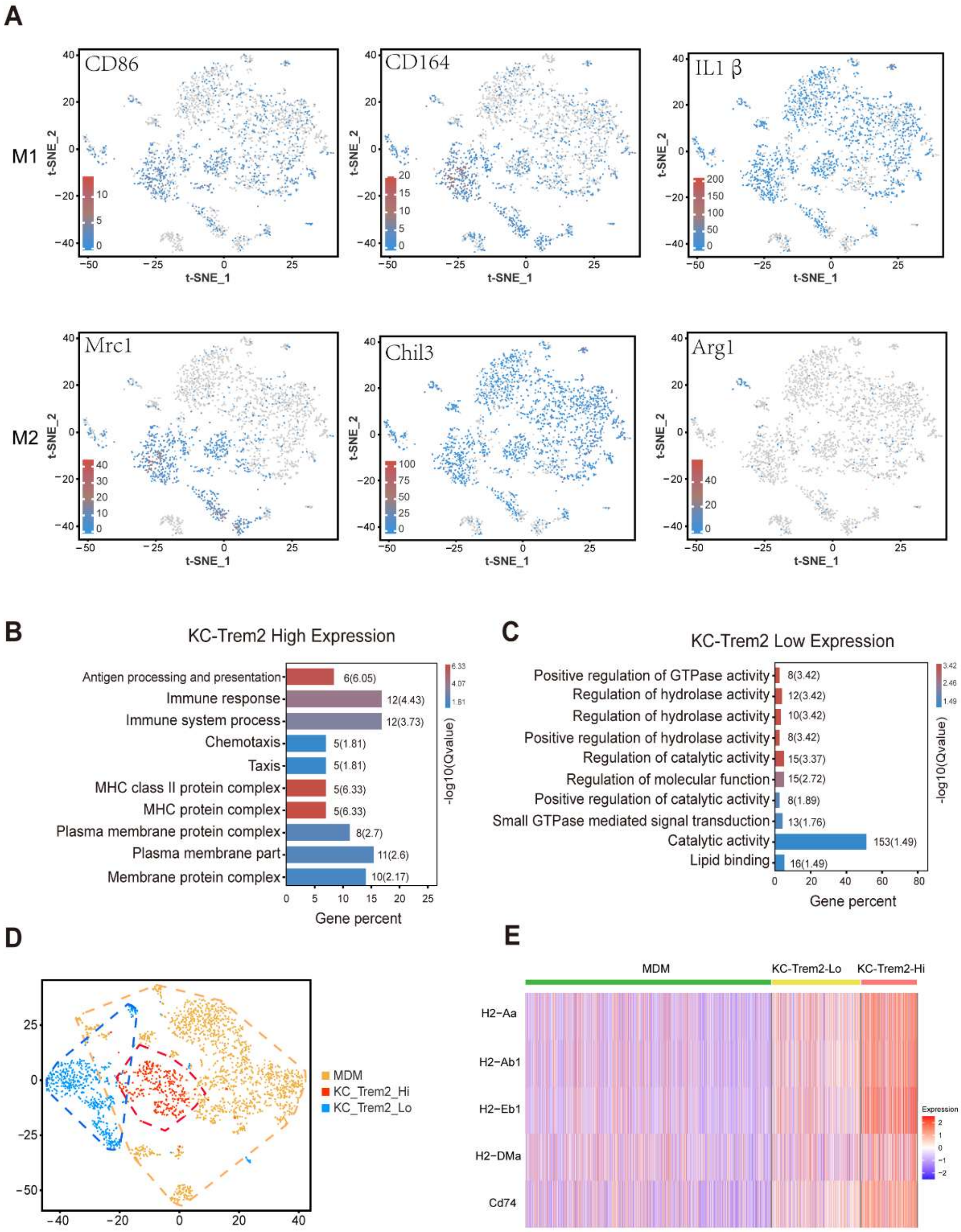

Figure S5 Hepatocyte-specific ERAD deficiency induces changes in T lymphocyte sub-types. (A-C) t-SNE plots based on separated WT (A) and HKO (B) and integrated (C) scRNA-seq data showing the defined T lymphocyte subtypes. (D) Stacked bar chart showing relative abundances of the defined T lymphocyte subtypes in WT vs HKO liver.

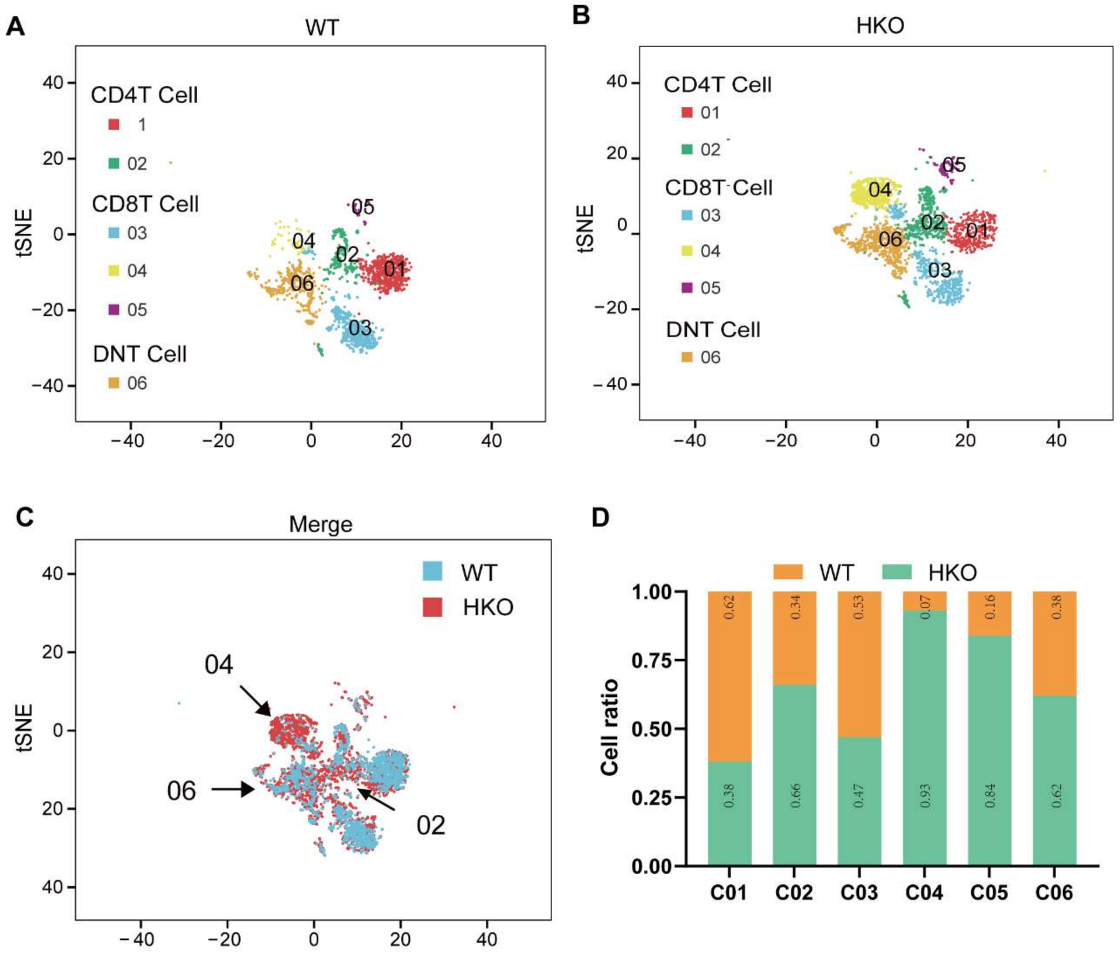

Figure S6. Short-term administration of CD8α mAb does not alter the abundance of total hepatic immune cells and macrophages. (A-G) FACS gating strategies (A, C, E) and corresponding quantification (B, D, F, G) of total immune cells (A-B), macrophages (C-D), Kupffer cells (F) and myeloid-derived macrophages (G) in IgG and CD8α mAb administered mice. (H-M) Body weight (H), liver to body weight ratio (I), qRT-PCR analysis of hepatic expression of proinflammatory genes (J-M) ($n = 6$ per group). (N) Liver tissue section from 3-month-old mouse. Black arrows: monocytes, red arrows: Councilman body ($n = 3$ per group). (O) Liver sections from 3-month-old mice were stained for αSMA by immunohistochemistry; black arrows indicate αSMA-positive cells ($n = 3$). (P) Percent of Councilman body. (Q) Number of mononuclear cells per 1000 nuclei. (R) Number of αSMA cells per 1000 nuclei. All data are presented as means ± SEM. ns, not significant; *$p < 0.05$, **$p < 0.01$, ***$p < 0.001$, ****$p < 0.0001$ by two-tailed Student's t test.

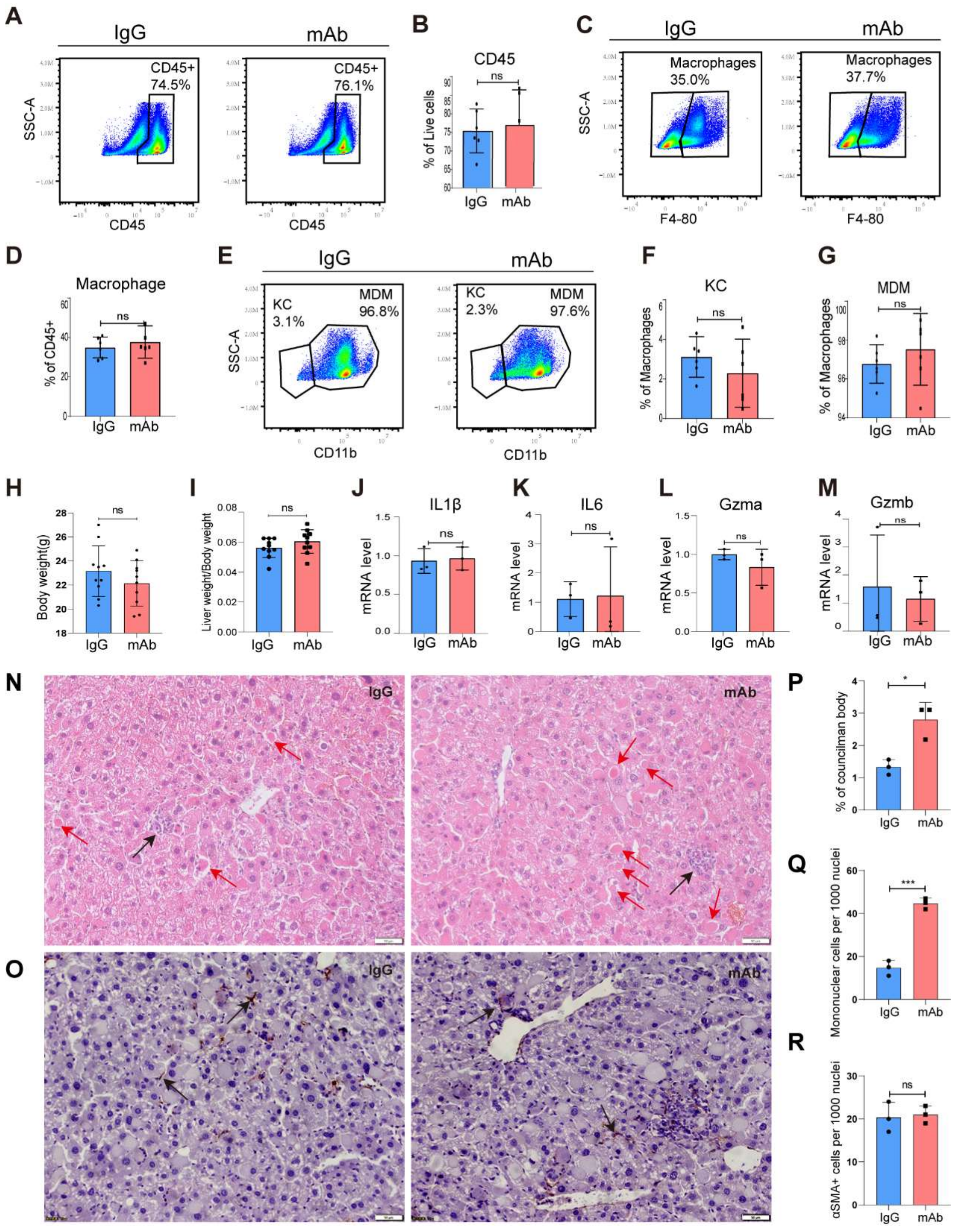

Figure S7. Flow cytometry gating strategy. Flow cytometry data were analyzed using a hierarchical gating strategy. Debris and doublets were excluded based on FSC-A/SSC-A and FSC-H/FSC-A plots, respectively, and dead cells were removed by staining with the Fixable Viability Dye eFluor™ 780. CD45+ cells were identified as leukocytes and analyzed using three parallel gating branches. F4/80+ cells were classified as total macrophages and subdivided into Kupffer cells (KCs, CD11b⁻) and myeloid-derived macrophages

(MDMs, CD11b+). CD11c+ cells were classified as dendritic cells (DCs) and activated DCs (MHCII+). CD19+ cells within the CD11b- fraction were identified as B cells, whereas CD3+ cells within the CD19- fraction were identified as T cells, which were further subdivided into CD4+ and CD8+ T cells. Within the CD11b+ fraction, Ly6C+ and Ly6G+ cells were identified as monocytes and granulocytes, respectively. Gates were set based on the fluorescence distribution of the corresponding negative populations and applied uniformly to all samples. Data were analyzed with FlowJo v10.8.1.

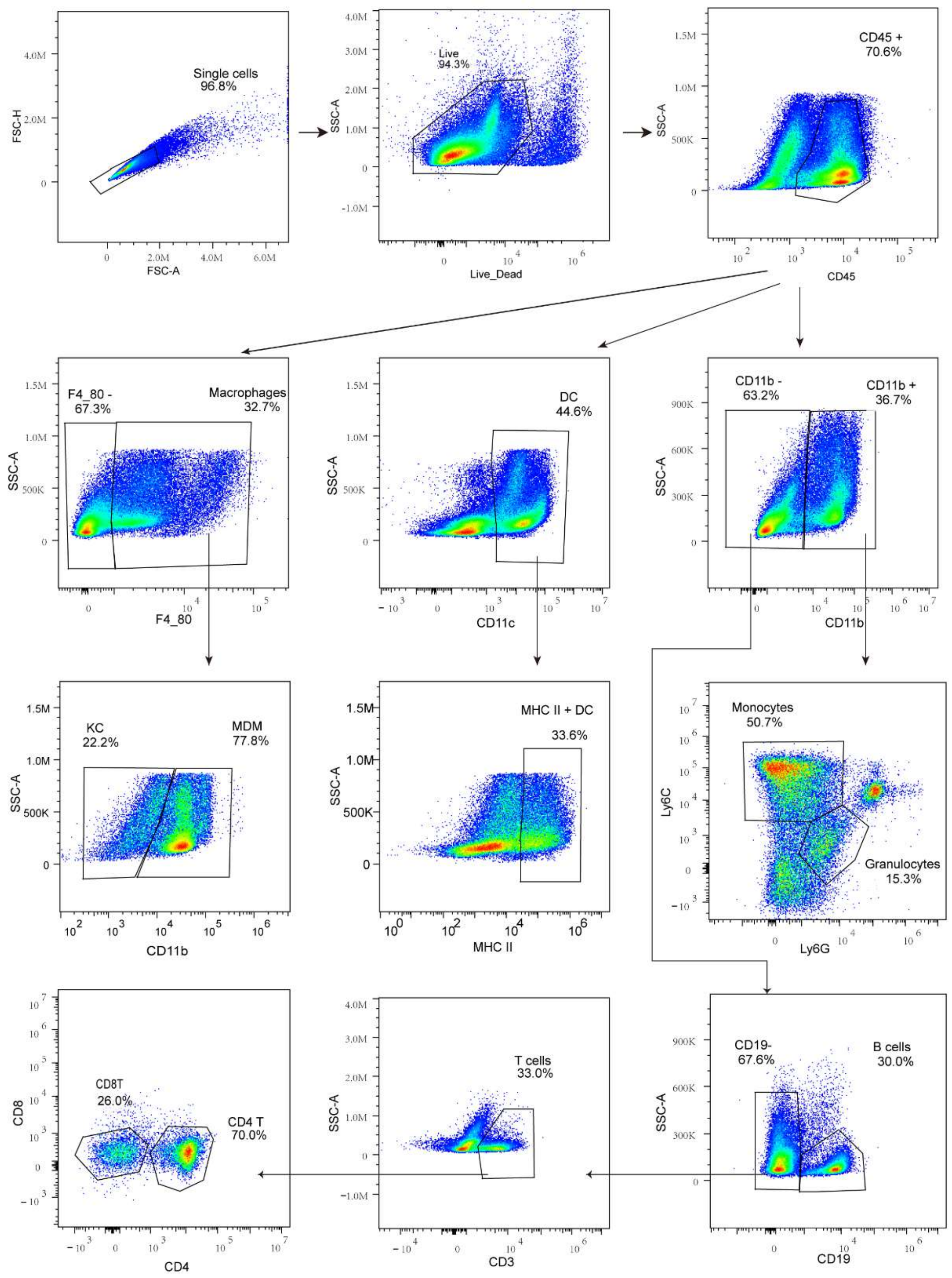

Table S1 Marker genes used for T cell subset identification. Hepatic T cells from integrated WT and HKO scRNA-seq data were clustered into six subsets (01-06). These six clusters were defined as 01-CD4T, 02-CD4T, 03-CD8T-Naïve, 04-CD8T-Effector, 05-CD8T-Exhausted and 06-DNT (double-negative T cells), respectively, based on marker gene expression. Values indicate the mean normalized expression of each marker in the corresponding subset. Colors reflect relative expression levels.

T cell marker	Gene_name	01-CD4T	02-CD4T	03-CD8T-Naïve	04-CD8T-Effector	05-CD8T-Exhausted	06-DNT
ENSMUSG00000032094	Cd3d	8.33694153	9.26199937	10.11488769	15.02444172	5.004603571	10.44198494
ENSMUSG00000032093	Cd3e	4.758565	6.133528852	5.455896504	13.65151791	3.037944388	6.586010051
ENSMUSG00000002033	Cd3g	6.096683156	8.038618845	6.436743006	17.71438597	4.314609689	9.167159757
ENSMUSG00000026012	Cd28	3.45782781	3.901634776	1.850401474	1.825941642	1.372750645	2.560100236
ENSMUSG00000053977	Cd8a	0.046228748	0.257947441	4.659862075	8.679643568	1.321164505	0.802828343
ENSMUSG00000023274	Cd4	2.227275609	2.501790949	0.025039793	0.406978738	0.316807096	0.497713572
ENSMUSG00000039521	Foxp3	0.008588578	1.085557213	0.001412113	0	0.159752271	0
ENSMUSG00000025163	Cd7	0.373196695	0.809304725	1.515516818	9.402259352	2.68702852	3.74871232
ENSMUSG00000024910	Ctsw	0.268924225	1.084099805	2.434162571	2.041030386	1.391331686	4.521829757
ENSMUSG00000075010	AW112010	1.947252665	13.54957483	8.261359735	45.63275469	14.78114043	19.32073638
ENSMUSG00000048251	Bcl11b	4.932244293	3.954982767	3.124283807	3.525006657	1.133887828	2.397286533
ENSMUSG00000057329	Bcl2	3.370318865	2.004324711	3.110791157	6.355309297	0.647122639	5.335523648
ENSMUSG00000035042	Ccl5	0.20729113	14.47433462	49.71799005	484.7505231	35.04003421	42.4882952
ENSMUSG00000030336	Cd27	1.599342366	1.450381006	1.502029343	3.021281771	0.713610122	1.468691923

Naïve cell marker	Gene_name	01-CD4T	02-CD4T	03-CD8T-Naïve	04-CD8T-Effector	05-CD8T-Exhausted	06-DNT
ENSMUSG00000027985	Lef1	8.039262475	1.199598608	4.039277047	0.179873883	0.297801377	1.450551547
ENSMUSG00000026581	Sell	5.038841456	1.18175353	3.998831046	0.133779519	0.465119077	0.591292773
ENSMUSG00000000782	Tcf7	7.19274678	3.345592502	5.01946993	0.163375154	0.715973944	1.515099609
ENSMUSG00000045092	S1pr1	4.003070312	2.276245987	2.268530423	0.227294642	0.654468949	0.594161612

Effector cell marker	Gene_name	01-CD4T	02-CD4T	03-CD8T-Naïve	04-CD8T-Effector	05-CD8T-Exhausted	06-DNT
ENSMUSG00000042385	Gzmk	0.003929821	0.143468543	0.308634287	6.251170755	1.303864033	0.057016865
ENSMUSG00000005087	Cd44	0.247431572	2.140396744	0.506514545	0.571830514	1.04228038	2.02085625
ENSMUSG00000023132	Gzma	0.048460469	0.229611547	1.14835344	23.52958593	4.672862841	3.419967939
ENSMUSG00000024778	Fas	0.323755939	0.371133095	0.267879443	0.101362203	0.188173085	0.197786404
ENSMUSG00000015437	Gzmb	0.010223422	0.179024435	0.728427676	13.89644389	2.302053761	3.041052751
ENSMUSG00000000817	Fasl	0.070921412	0.311119081	0.235977707	1.757442002	0.159371298	1.026679094
ENSMUSG00000003882	Il7r	7.373149871	8.597104417	6.506902878	0.215387226	0.534490024	4.352735516
ENSMUSG00000055170	Ifng	0.010072269	0.383048053	0.219359991	0.890660502	0.351394589	0.940932763

Exhausted cell marker	Gene_name	01-CD4T	02-CD4T	03-CD8T-Naïve	04-CD8T-Effector	05-CD8T-Exhausted	06-DNT
ENSMUSG00000026285	Pdcd1	0	0.288796721	0.060828251	3.581373561	0.418842153	0.036735654
ENSMUSG00000030124	Lag3	0.008861383	0.048296999	0.107263312	2.894474855	0.246129788	0.08142529
ENSMUSG00000026011	Ctla4	0.173088055	2.214150068	0.257373057	1.647401252	0.891132829	0.041549621
ENSMUSG00000071552	Tigit	0	0.046626534	0.004396578	0.210863222	0.032213809	0.008655469
ENSMUSG00000000782	Tcf7	7.19274678	3.345592502	5.01946993	0.163375154	0.715973944	1.515099609
ENSMUSG00000015437	Gzmb	0.010223422	0.179024435	0.728427676	13.89644389	2.302053761	3.041052751
